# Avoiding potential pitfalls in visual search and eye-movement experiments: A tutorial review

**DOI:** 10.3758/s13414-021-02326-w

**Published:** 2021-06-04

**Authors:** Hayward J. Godwin, Michael C. Hout, Katrín J. Alexdóttir, Stephen C. Walenchok, Anthony S. Barnhart

**Affiliations:** 1 School of Psychology, University of Southampton, Highfield, Southampton, Hampshire, SO17 1BJ UK; 2 Department of Psychology, New Mexico State University, Las Cruces, NM USA; 3 Department of Psychology, National Science Foundation, Alexandria, VA USA; 4 Department of Psychology, Arizona State University, Tempe, AZ USA; 5 Department of Psychological Science, Carthage College, Kenosha, WI USA

**Keywords:** Eye movement, Visual search, Tutorial review

## Abstract

Examining eye-movement behavior during visual search is an increasingly popular approach for gaining insights into the moment-to-moment processing that takes place when we look for targets in our environment. In this tutorial review, we describe a set of pitfalls and considerations that are important for researchers – both experienced and new to the field – when engaging in eye-movement and visual search experiments. We walk the reader through the research cycle of a visual search and eye-movement experiment, from choosing the right predictions, through to data collection, reporting of methodology, analytic approaches, the different dependent variables to analyze, and drawing conclusions from patterns of results. Overall, our hope is that this review can serve as a guide, a talking point, a reflection on the practices and potential problems with the current literature on this topic, and ultimately a first step towards standardizing research practices in the field.

**Supplementary Information:**

The online version contains supplementary material available at 10.3758/s13414-021-02326-w.

**Keywords:** Visual search; Eye tracking; Tutorial review

## Introduction

Visual search is an important task that is required for our success in a wide range of everyday activities. From searching for a mug of coffee on our desk, to searching for an app to open on our phones, or searching for our cat in the garden, the visual searches that we engage in typically require eye movements in order to find our targets. Search also plays a key role in many societally important tasks, such as when radiologists search for tumors (for a review, see van der Gijp et al., 2017) or when X-ray screeners search for threats (for a review, see Donnelly et al., 2019). Additionally, visual search is used as a tool by researchers to study a broad spectrum of different “basic” aspects of cognition and information processing, and, accordingly, researchers have used a wide array of different approaches and metrics to understand visual search (for reviews, see Eckstein, 2011; Rayner, 2009).

Here, we review one very popular approach that has been used to better understand cognition and information processing during visual search: the recording of eye-movement behavior. Eye movements are made up of fixations (where the eyes are relatively still) and saccades (where the eyes make rapid, ballistic motions, with *saccade* being the French word for “jerk”). Given the limited resolution of peripheral vision, eye movements are required to bring the high-resolution fovea to bear upon objects or areas of interest within our environment, allowing us to examine, in detail, those objects or areas of interest. We make, on average, about four to five eye movements every waking second. Several researchers have argued that eye movements are the most common of all behaviors that we engage in (Bargary et al., 2017), and there is some evidence that we each have idiosyncratic patterns of eye-movement control, much like a signature or unique “fingerprint” of our own information-processing (Arizpe et al., 2017; Poynter et al., 2013).

**Fig. 1:**
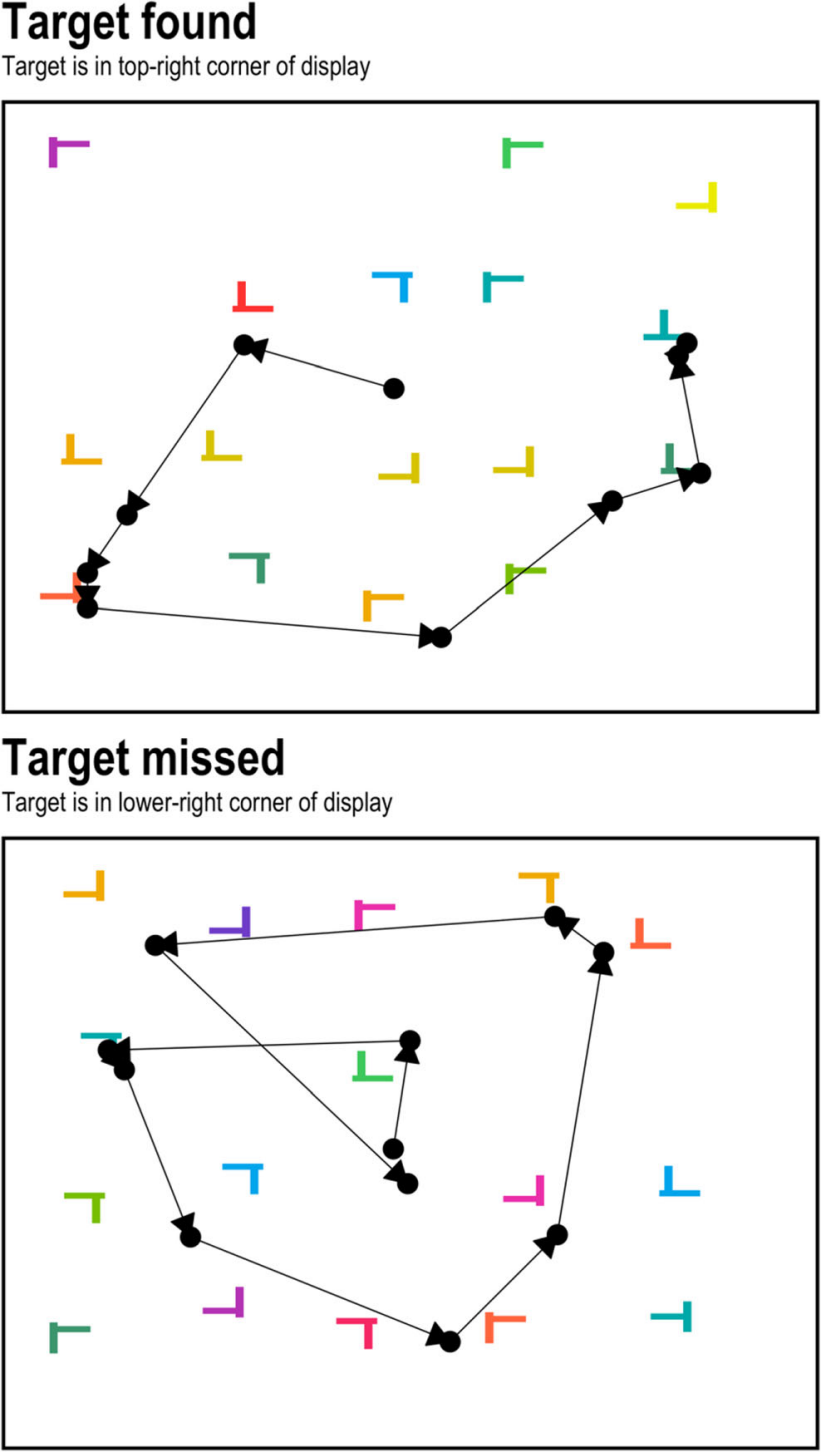
Two example visual search trials. *Note:* In this experiment, the participant was searching for two “T” shapes, but only one target could appear per trial. Each point is the location of a fixation during visual search. Data are from a previous set of experiments (Godwin, Menneer, Riggs, et al., 2015)

A set of two example trials from a visual search task, along with where the eyes fixated, are presented in Fig. 1. The two examples are drawn from the same participant and represent one trial where a target (a teal T-shape) was detected after being fixated (top panel), and a second trial where a target was not fixated and not detected (lower panel). This is just one instance where the recording of eye-movement behavior can help to provide deeper theoretical insights than the recording of response times and response accuracy alone. Without eye tracking a researcher would be unable to determine specifically why an incorrect response was given in the lower panel. However, with eye tracking, we can resolve this question of why an incorrect response was given with ease: Put simply, the participant missed the target because they never looked at it (which can be contrasted with a situation in which the target was directly fixated but the participant failed to perceive it as the target).

Because eye tracking can provide unique insights into what takes place during visual search, it is regarded as an excellent measure of online, moment-to-moment information processing (for a number of reviews on eye movements as they relate to information processing both within and beyond the scope of visual search, see Liversedge et al., 2011). Although recording eye movements might not necessarily be useful in some paradigms (such as those utilizing briefly-presented search displays), eye movements can largely enrich studies of visual search through revealing subtle aspects of cognition beyond accuracy and response time. Eye-movement metrics can offer insights into object identification and attentional guidance in visual search; for example, Hout et al. (2015) showed that low-prevalence targets were often fixated and then marked as “target-absent” by participants; Walenchok (2018) extended the findings of Rajsic et al. (2015, 2017) by showing that when the temporal cost of searching through a larger subset of stimuli is high (i.e., with an overall large set size also containing a disproportionately large subset size of a specific color of stimuli), this encouraged an inferential search strategy, negating the need to necessarily inspect the target (i.e., all trials contained a target, and participants inferred the target color through exclusion).

**Fig. 2:**
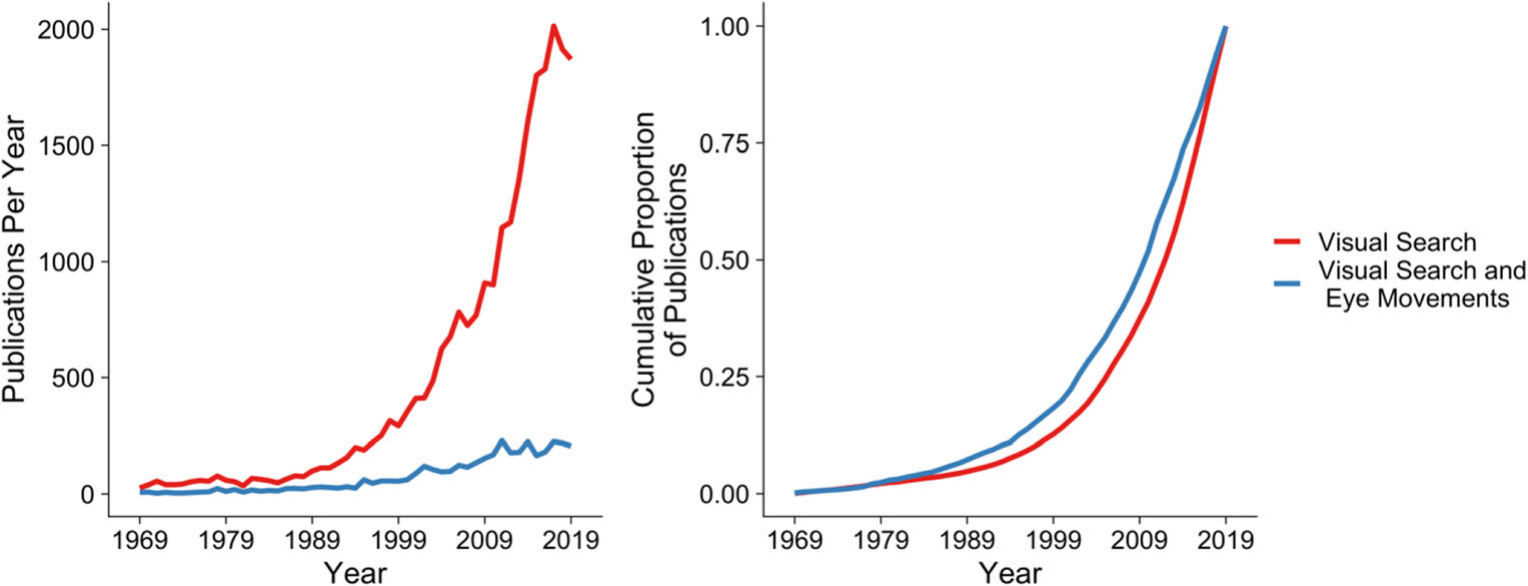
Fifty years of visual search and eye-movement publications. *Note:* To obtain the results for these graphs, we conducted a search using EBSCO spanning the years 1969–2019. For the “Visual Search” results, we conducted a search of abstracts and titles containing “visual search.” For the “Visual Search and Eye Movements” results, we conducted a search of abstract and titles containing “visual search,” “eye movement,” “eye-movement,” “fixation,” or “saccade”

It is perhaps not surprising, therefore, that there has been a steady but clear increase in the number of visual search and eye-movement articles during the past half-century (see Fig. 2). Alongside the unique theoretical insights that eye-movement recordings can offer, it is worth noting that the rise in popularity is likely to also be due to technological improvements and widening access to those improved forms of technology (for an historical review, see Wade, 2010). For example, although the earliest studies of visual search and eye movements date back to the work of early pioneers such as Delabarre (1898) and Buswell (1935), even as recently as the late 1990s eye-movement behavior during visual search was recorded using scleral coils (Findlay, 1997; Hooge & Erkelens, 1996). This involved fitting a metal coil to each participant’s eye, and the procedure was sufficiently uncomfortable that participants needed to have their eyes anaesthetized in order to take part in experiments (Eggert, 2007). Modern video-based eye-trackers do not involve such a high level of discomfort for the participants, and it is now possible to record a participant’s eye-movement behavior while they rest their head in a chin rest whilst a camera records their eye movements using non-invasive, near infrared light. In fact, technological advancements now mean that most of us carry eye-trackers around with us every day, as it is possible to use the front-facing cameras of cellphones as (albeit primitive) eye trackers (see, e.g., Drewes et al., 2007). Although covering the advantages and disadvantages of different eye-tracking systems is outside the scope of this review, higher-end eye-tracking systems such as those from SR Research (www.sr-research.com) and Tobii (www.tobii.com) as well as more affordable options such as those from Pupil Labs (pupil-labs.com) offer the ability to record high-quality eye-movement data, depending on the needs of the researchers (e.g., required accuracy and sample rate, head-mounted vs. desk-mounted cameras, and so forth).

However, just because the barriers to recording eye movements have been steadily reducing over the last 50 years, it is not necessarily the case that developing a well-designed eye-tracking study is easier now than it used to be. As we shall see throughout this tutorial review, there are a variety of highly complex and important decisions made by researchers – sometimes, without them realizing – that arise throughout the research cycle when it comes to engaging in visual search and eye-movement studies. Because of this, there are increasing concerns that, throughout the various studies of eye tracking, researchers often make a number of systematic errors in their approach: errors that render their reported results at best plagued by statistical noise, and at worst, entirely incorrect or uninterpretable (Orquin & Holmqvist, 2018).

The goal of the present review, then, was to provide a walkthrough of the research cycle that one may go through when conducting a visual search and eye-movement experiment. The walkthrough runs from the start of a research project (theoretical underpinnings), through to methodological decisions such as the choice of eye tracker, moving on finally to the cleaning and analysis of datasets and how to draw conclusions from the analyses. Throughout, we focus on lab-based visual search tasks as opposed to those that involve the use of head-mounted eye trackers during search, because these typically require different methods than those discussed here (e.g., Riggs et al., 2018). As we progress through the tutorial, we adopt an approach that has become increasingly popular in reviews of this type (e.g., see Godfroid & Hui, 2020) by highlighting potential pitfalls that could cause problems for researchers at each stage of the research cycle. Hereafter we refer to these “potential pitfalls” as “pitfalls.” We do this because even if the steps to avoid the pitfalls were taken but not reported, we have no way of knowing this one way or another based on reading the papers. Moreover, if certain methodological or analytical steps are carried out but not reported, the issue then is a different one involving a failure to be transparent and fully report all the required information in published research. To put this another way, the pitfalls we highlight could act as threats to validity or could limit reproducibility and replicability if authors fail to explicitly note how (or if) the pitfalls were addressed in their designs and analyses. Ultimately, a number of the pitfalls could be curtailed through thoughtful open science practices like preregistration and thorough description of analytical strategies.

## A basic framework for understanding eye movements during visual search

Before turning to our walkthrough, we briefly sketch out a basic framework for understanding the eye-movement behavior that takes place during visual search tasks. As already highlighted in Fig. 1, a searcher tends to fixate upon objects in turn until either a target has been found or is deemed to be absent. Some objects are fixated once, others are fixated multiple times. Not all objects are visited, yet some are revisited. Searchers tend to focus their fixations upon objects that share visual or semantic similarity with the target(s) being searched for (Becker, 2011; Godwin, Hout, & Menneer, 2014; Godwin, Reichle, & Menneer, 2017; Hout & Goldinger, 2015; Luria & Strauss, 1975; Stroud et al., 2012), a finding that is much in line with behavioral models of visual search (Duncan & Humphreys, 1989; Wolfe et al., 1989). In addition, objects that share similarity with the target(s) being searched for tend also to be fixated for longer than objects that are less similar to the target(s) (Stroud et al., 2012).

Although one might assume that the entirety of a fixation’s duration is required to process the identity of the fixated object, this is not likely to be the case. One interesting demonstration of this is that, in a previous study, it was found that removing a search target from view after only between 0 and 9 ms reduced participants’ *confidence* that they have found the target but not their *accuracy* (Kotowicz et al., 2010). Similarly, reading proceeded normally even when words were presented very briefly (50 ms) followed by a mask (see Rayner, 1998). If it is the case that only a small amount of time is required to examine an object (or word) during a fixation, then why are fixations during search typically around 160 ms^1^ (in the case of Godwin et al., 2015)?

The simple answer to this question is that, although the visual system is acquiring visual information to process during a fixation, that is not all that takes place while the eyes are still. Alongside the acquisition of visual information, the next saccade (or possibly *saccades*) is/are being planned. The time to plan a subsequent saccade is known to follow a simple pattern. Biomechanical movements such as saccades are *ballistic:* once initiated, they cannot be terminated beyond a given deadline. Like any physical movement, they also require time to plan and initiate the underlying movements within the musculature. It is generally believed that there is a close relationship between the time required to plan a saccade and the distance that the planned saccade needs to travel, with more time being required for the eyes to traverse a greater distance (Bartz, 1962; Hackman, 1940; Kalesnykas & Hallett, 1994). Therefore, during a fixation, the visual search system not only picks up information from the object being fixated, but also prepares a saccade to the next location. Put another way, eye movements during search involve *asynchronous parallel processing* of both visual information and saccadic planning. Eye movements during reading also involve similar parallel processing, with saccade planning for word *n*+1 occurring during fixation of word *n* (Reichle et al., 2003). An asynchronous approach of this nature allows the visual system to be more efficient. Operating in this asynchronous manner reduces the “dead time” that would occur if the system waited for visual information to be fully processed before even beginning to plan the next saccade (Hooge & Erkelens, 1996).

But there is still more to the story. Previous research has shown that planning to fixate a given location facilitates processing of visual information at that location (Deubel & Schneider, 1996). What this also means, therefore, is that as part of the saccadic planning process, the search system can also begin to *pre*-process the identity of the next object that will be fixated (a notion that neatly dovetails with behavioral models of visual search that have suggested the same idea; see Townsend, 1971, 1990; Wolfe, 1998). Perhaps more surprisingly, it has also been shown that removing the entire search array from view does not necessarily stop searchers from processing the identities of the objects after they have been removed from view. In fact, removing the entire search array from view during an in-process search merely slows searchers who continue to fixate locations where objects *had* been located (Godwin et al., 2013). These objects are generally not returned to after the display is reinstated, suggesting that search can continue to process the identities of absent objects based entirely on the information that had been pre-processed prior to direct fixation.

It is unclear how far ahead the search system plans in terms of future fixations, or whether this planning process is limited by, for example, the complexity of the stimuli as well. Gilchrist and Harvey (2006) note that a fixation plan that encompasses all objects within a display would preclude the need for remembering which objects had already been inspected. Instead, all that would be needed would be a record of where in the fixation plan the searcher was currently fixating. Turning this issue on its head, it is also unclear as to exactly how extensive the memory record is for already-inspected objects in visual search. Typically, researchers use revisits to already-inspected objects as an indicator that the searcher had “forgotten” that they had already inspected the objects in question (e.g., see Peterson et al., 2001). Although this is a logical supposition, the problem with this as an assumption is that the majority of revisits to already-inspected objects take place immediately after the eyes first leave the object (Engel, 1977; Gould, 1967; Hooge & Erkelens, 1996; Peterson et al., 2007; Wienrich et al., 2009). For example, suppose that a searcher visits object *n* and then moves to object *n+1*. A brief period of time elapses and they then return to object *n*. These so-called *lag-2 revisits* are very common during visual search, and account for the majority of the revisits that take place. It is unlikely that they are the result of the searcher having forgotten that they just examined the already-inspected object; rather, it is generally accepted that these lag-2 revisits occur because a fixation is prematurely terminated on object *n* before it has been fully processed, and the brief fixation on object *n+1* is brief simply because it is terminated rapidly in order to allow the search system to return to object *n*. For the most part, these lag-2 revisits occur for target objects, further suggesting that they are willful re-inspections, rather than random errors in saccadic targeting (see also Godwin, Reichle, & Menneer, 2017). Moreover, the brief period of time spent on object *n+1* further suggests that the search system made a corrective saccade, much like a correction for a targeting error, back to object *n*. Note that similar refixation phenomena also occur during reading, wherein revisits can reflect corrective saccades, incomplete lexical processing, or contextual factors such as less predictable words (Rayner, 1998).

In the current section, we have provided details regarding some of the complexities of what movements our eyes make during visual search. When searching, an individual will focus on objects that share some similarity with the targets, fixating them in turn until a target has been found or is deemed to be absent. Not all objects will be fixated, and some will be fixated longer than others. Some objects will be revisited, and the most likely time for a revisitation will be as part of a lag-2 revisit, and these lag-2 revisits will primarily occur for target objects. Finally, failing to detect a target – an occurrence seen in Fig. 1 – is likely to occur when the target is not directly fixated. The basic framework presented here has, by necessity, been quite brief: More detail can be found in various models of visual search and eye movements (e.g., see Hulleman & Olivers, 2017; Zelinsky, 2008).

## A walkthrough of the research cycle of a visual search and eye-movement experiment

In this section, we provide a walkthrough of the research cycle for visual search and eye-movement experiments, highlighting potential pitfalls along the way. For this walkthrough, we draw data, evidence, and illustrations from several different sources. Before beginning the walkthrough, we describe each of these sources in turn.

### Brief Review

In order to provide some indication of how widespread the pitfalls that we highlight might be, and to illustrate how visual search experiments are typically conducted and analyzed in the literature, we performed what we hereafter refer to as our brief review of some of the existing literature that has examined visual search and eye-movement behavior. We began by searching for the same search terms as in Fig. 2, but narrowed the search down in two key ways: First, we limited the search to focus on papers published in *Attention, Perception, & Psychophysics;* second, in order to gain insights into the current research zeitgeist, rather than an historical one, we limited the search to focus on papers published between 2014 and the time of writing this review (2020). This *Brief review* was not intended to serve as a full systematic review, but rather to aid us in gaining some insights into the scope of the pitfalls as we discuss them.

The initial search returned a total of 51 items. There were no duplicates returned as part of this search. We excluded items that (a) did not present primary research, (b) did not analyze any eye-movement-dependent variables, (c) did not only recruit healthy human adults as participants, or (d) used dynamic displays (e.g., videos). After removing items for these reasons, the final set comprised 20 published papers. Together, these published papers presented results from a total of 37 experiments and 1,083 participants. A full set of interactive tables relating to this activity, along with direct links to the papers themselves (many of which are open access) can be found in the Online Supplementary Materials (OSM).

### Visual search paper

To illustrate our discussion with real data, we present data from an already-published set of experiments (Godwin et al., 2015).^2^ We have already seen example trials from this experiment, presented above in Fig. 1. These experiments were published in a single paper and share some similarity with those captured by our *Brief review*: the paper was also published in *Attention, Perception, & Psychophysics* and involved visual search and eye-movement behavior. For that reason, it serves as a talking point given its similarity to the other papers that we reviewed. In both experiments reported, participants searched for two targets. Both targets were T shapes and the distractors were L shapes. A target appeared in 50% of trials, but one of the targets appeared in 45% of trials, whilst the other appeared in 5% of trials (Godwin et al., 2010; Hout et al., 2015). Both experiments sought to better understand the manner in which rare targets are missed during visual search (Wolfe et al., 2005, 2007) – a phenomenon known as the “prevalence effect” – and we talk more about this in detail as the review progresses. The data from both of these experiments, and the R code associated with the walkthrough, are all available online at https://osf.io/2nmtc/.

### Terminology

Even for a veteran eye-movement researcher, one problematic issue can be the adoption of the correct (and consistent) terminology to describe the basic behaviors, concepts, and approaches under examination. In recent reviews of pitfalls associated with the study of other visual cognitive tasks and eye movements (Godfroid & Hui, 2020; King et al., 2019), it has been noted that there is a rather dizzying array of different terms used to describe the same concepts, behaviors, and dependent variables. Elsewhere, following a large survey of eye-movement researchers, the authors of the survey argued that researchers are “confused” regarding how to define fixations and saccades (Hessels et al., 2018). Although we do not wish to retread this ground, we believe that the same problem exists within the literature involving visual search and eye movements.

For the sake of consistency and clarity, here we define the basic units of eye-movement behavior, fixations and saccades, as described above. When the eyes land on an object during a search, that begins a *visit* on that object (elsewhere, visits have been called *gazes, glances,* and/or *dwells*). All of the fixations that land upon that object (until either a new object is fixated or a fixation falls far from that object) are classified as one visit upon that object. When the eyes return to that object after visiting elsewhere, a new visit begins. Other researchers will certainly take different approaches to this same terminology, and regardless of which terminology is used in the future, we believe that a useful first step will be for all researchers to define the terminology that they use in each paper.

### Choosing the right predictions

Having outlined a basic framework for understanding eye-movement behavior during visual search, we can now begin our walkthrough of the research cycle. The first step in the research cycle involves choosing an appropriate experimental design, as well as making appropriate predictions. Before this, of course, one needs a theory on which to ground the research questions and predictions being investigated. In the context of Godwin et al. (2015), previous research into the effects of low target prevalence at that time had, for the most part, not involved eye-movement recordings. At that point in time, models of the “prevalence effect” in visual search had focused largely on behavioral data (e.g., reaction times (RTs) and accuracy), leading to the development of the Multiple Decision Model (MDM) of search (Wolfe & Van Wert, 2010).

The MDM is worth discussing in detail here because it provides a talking point that can serve as a brief overview of many models of visual search. It builds upon previous models, such as Guided Search (Wolfe et al., 1989), and assumes that during search, people tend to focus on objects that are similar to the target being searched for. As part of this model, search behavior is broken down into a series of two-alternative forced-choice decisions. In the first of these, for each object examined during search, the search system asks the question *Is this object the target?* If the answer is “yes,” then a “present” response is generated and the search ends. If the answer is “no,” then the searcher will continue, but only up to a certain point. That point is addressed by the second question: *Should I quit searching?* The search system will answer “yes” to this question when an internal threshold that dictates how long an unsuccessful search should last is reached. The threshold is set adaptively based on how likely it is that a target will be present (Chun & Wolfe, 1996). The search system will otherwise answer “no” when the internal timer to quit searching has not been reached. Although it is tempting to assume that the search system only examines one object at a time, it has long been known that several objects can be processed in parallel by searchers (Townsend, 1990; Wolfe, 1998). This model can be rather straightforwardly translated into eye movements and visual search behavior if one assumes that the object that the searcher is making a decision about for the first of the two decisions is the one currently being fixated (Wolfe, 2020).

In the experiments conducted as part of Godwin et al. (2015), the goal was to examine aspects of this model more closely, and better understand *how* and *why* low-prevalence targets are missed during visual search. For example, we wanted to ask: were rare targets missed because they were rarely looked at during search? Alternatively, were rare targets fixated, but then, due to a failure in object identification processes, still missed? Answering these two questions is not possible without eye tracking, and for that reason, monitoring participants’ eye-movement behavior was an ideal route to enriching existing theories. We predicted that rare targets would be more likely to be missed by searchers due to a combination of searchers (a) failing to fixate targets and (b) missing targets even after fixating them. In general, our results upheld these conclusions, with a set of predictions derived from an existing model of visual search (at the same time, a near-identical set of questions were also being addressed by Hout et al. (2015), with the results of the two sets of studies mirroring one another).

The MDM was able to generate predictions for eye tracking based on behavioral results thanks to an existing model of the basic behavior being examined. Unfortunately, such models are not always available in the context of generating predictions for a new study. Worse still, researchers often fall into a simple but altogether too common trap: By failing to acknowledge the complex underpinnings of oculomotor control, they can run an experiment wherein a difference between conditions is *expected* regardless of any experimental manipulations at work. The dangerous outcome of this is that when researchers do find a significant difference between their experimental conditions, they conclude that the significant difference is the result of there being a true underlying difference in the data and in human behavior. However, in fact, the difference may emerge because the researchers have failed to account for a broader spectrum of issues and tendencies in how eye movements are controlled, and in reality there is no true difference to be found.

In a recent review and critique, Flake and Fried (2020) discuss what they term *Questionable Measurement Practices* (QMPs). These QMPs, they argue, arise from a relaxed attitude towards a detailed, well-documented, and formalized set of variables and measurements in psychological science (they call this a “measurement-schmeasurement” attitude). In this section, we describe some instances where confounds and problems with *choosing the right predictions* can occur, and how they can give rise to QMPs at the earliest stage of a study’s development process such that a study is virtually guaranteed to draw incorrect, unreliable, or confounded conclusions before a single eye movement is recorded or before the study has even been programmed and tested.

#### Pitfall #1: Failing to consider visual similarity when generating predictions or designing a study

When conducting an eye-movement experiment, one can, for example, assume that participants should spend an equal amount of time inspecting two different objects, and that any differences in the time taken to inspect those objects can *only arise* from an experimental manipulation (i.e., the independent variable(s)). However, the differences in the time taken to inspect those objects might be *expected* simply upon the basis of differences in the visual characteristics of those objects alone. For that reason, it is worth noting the importance of ensuring that, in any study, a researcher is not failing to compare “like for like.”

In a previous review of ‘threats to validity’ in examining eye-movement datasets (Orquin & Holmqvist, 2018), it was noted that there were a number of instances in the literature where this issue may have become manifest. They cite a study conducted by Baker et al. (2013) that involved comparing eye-movement behavior when participants were presented with images of neurological scans versus bar graphs, seeking to understand whether neurological scans are intrinsically more interesting or better capture attention than bar graphs. In this instance, Orquin and Holmqvist (2018) argue, it is difficult to make a fair comparison between these two disparate types of images since they differ so substantially in their visual properties.

The only acceptable route forward for a study of this type would be to quantify the visual properties between different objects, as well as how similar or dissimilar they might be, and take this quantification process into account when comparing eye-movement behavior in relation to those objects (we noted the importance of visual similarity earlier in this review). A number of studies now have used multidimensional scaling (MDS) to directly assess the visual similarity of different objects (for a review, see Hout et al., 2016). An example of this comes from a line of research investigating the role of semantic similarity in guiding search. Although it was initially believed that semantic information does not guide visual search (Wolfe & Horowitz, 2004), more recent investigations have suggested otherwise. One instance of this approach comes from a previous study where we quantified the visual similarity between numbers during a visual search for a specific digit (Godwin, Hout, & Menneer, 2014). When examining eye-movement behavior, we were able to account for the effects of semantic similarity (defined in terms of the numerical distance between the target number and those in the display), as well as the visual similarity of the numbers in order to draw conclusions from the eye-movement behavior. Not surprisingly, participants were likely to fixate distractors that were visually similar to the search target. However, they were also more likely to fixate distractors that were numerically similar to the target. While this work focused on the probability of fixating distractors, subtly different research questions could have led to examination of more fine-grained metrics of search behavior. Presumably, similar distractors would elicit longer dwell times, since they would be harder to reject than dissimilar distractors. Regardless, using quantifications of object similarity builds in the importance of visual similarity at the design level so that it does not confound a study at the point of analyzing the results and drawing conclusions.

In Godwin et al. (2015), we primarily compared metrics in terms of the high-prevalence target and the low-prevalence target. We quantified their visual similarity by using a set of colors for the targets and distractors based on previous work. This work created a perceptually uniform color space such that the colors could be represented in terms of “steps” from one another on a color wheel. For one experiment, the targets were one step in color from each other (i.e., they were visually similar), for a second experiment, the targets were eight steps in color from each other (i.e., they were visually *dissimilar*). This again helped us to avoid pitfall #1 by virtue of the fact that we built visual similarity into the design of the study: Indeed, differences were key in the study and the conclusions, rather than a confound. In general, the papers we examined as part of our *Brief review* did frequently show evidence of considering visual similarity when designing studies (85%), often by factoring in differences in the stimuli as part of their design.

#### Pitfall #2: Failing to consider coarse-to-fine behavior when generating predictions

In a similar vein to Pitfall #1 is another basic behavior relating to eye-movement behavior during visual search that needs to be considered when generating predictions. There have been several studies examining *coarse-to-fine* behavior in eye-movement datasets. Perhaps the first of these was an exploratory study conducted by Over et al. (2007).^3^ The authors observed that, as a visual search trial progresses, fixation durations tend to increase and saccade amplitudes tend to decrease. This was interpreted in terms of search beginning in a broad, “coarse” fashion, with briefer fixations distributed more widely across the display, which gradually become more fine-grained, with longer fixations and shorter amplitudes towards the end of a trial.

**Fig. 3:**
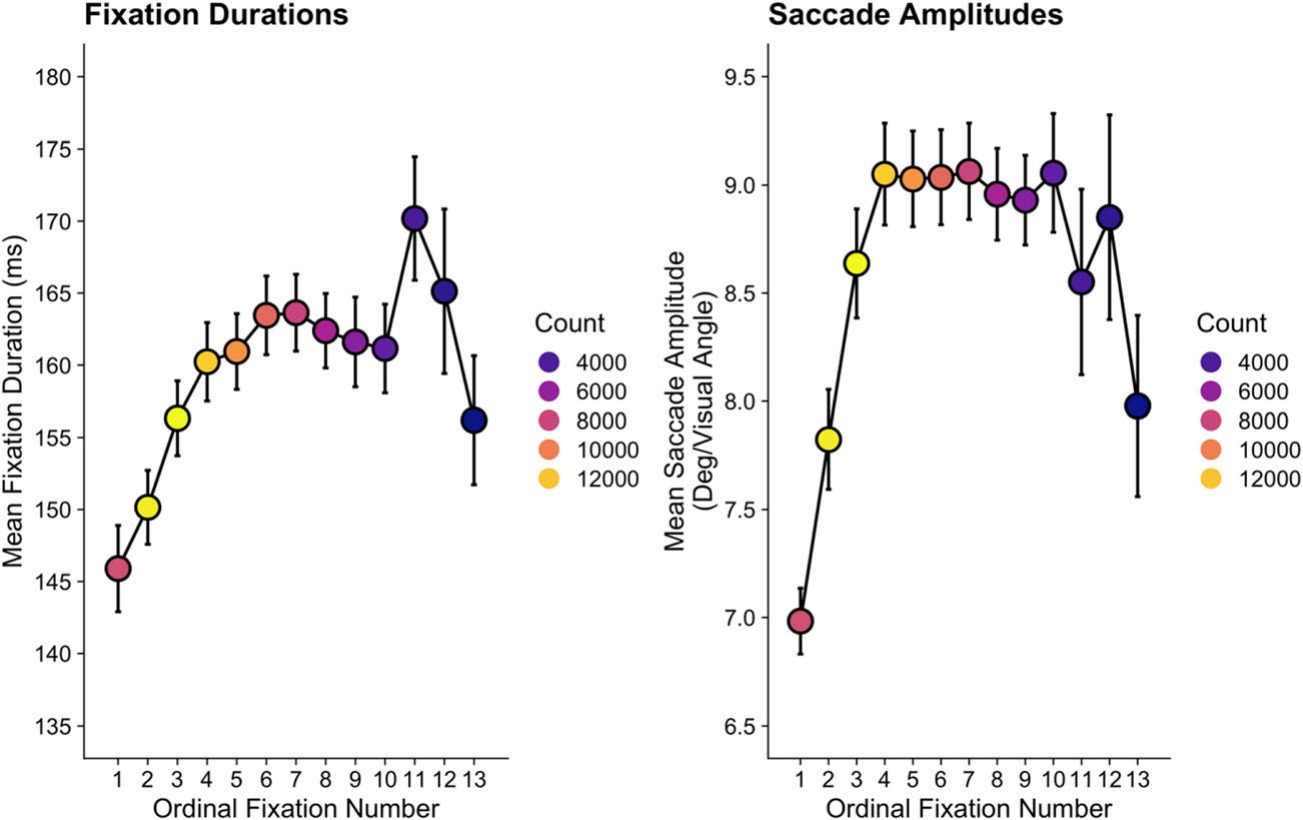
Coarse-to-fine behavior in a visual search study

We show coarse-to-fine behavior from Godwin et al. (2015) in the two panels of Fig. 3. One detail immediately worth noting is that saccade amplitudes increase, plateau, and then decrease, so a more accurate approach may be to use the less-eloquent term “fine-to-coarse-to-fine” behavior. A relationship between ordinal fixation number and both fixation durations and saccade amplitudes has also been reported in the scene perception literature (Castelhano et al., 2009; Cronin et al., 2020), suggesting that this behavior is not limited to simple visual search displays alone.

Despite the apparent universality of the behavior, Over et al.’s (2007) initial description of coarse-to-fine behavior as a “strategy” has not been upheld by all researchers. In a study and associated visual search model proposed by Godwin et al. (2014), it was noted that coarse-to-fine behavior could emerge from the basic mechanics of oculomotor control. In particular, towards the end of a search there will be fewer objects left unexamined, and these may be more widely distributed across a search display. As a consequence, a searcher will need to make longer saccades later in a trial compared to the beginning. These longer saccades require more time to program (Bartz, 1962; Hackman, 1940; Kalesnykas & Hallett, 1994), so that will cause an increase in fixation durations as more objects have been inspected and fewer objects remain to be visited. This, it was argued, could produce the observed coarse-to-fine behavior.

Regardless of the true underlying cause of coarse-to-fine behavior, it is relevant to researchers examining eye movements and visual search because it renders any comparisons of duration-based or amplitude-based metrics problematic when trials in different conditions have different numbers of fixations, which typically occurs when the trials are of different durations (i.e., when RTs differ). For example, in the context of studies wherein participants quit searching in one condition sooner than in another condition (a common occurrence in studies of low target prevalence that preceded the experiments conducted by Godwin et al., 2015), leading to a different number of fixations-per-trial in the two conditions, the prediction *should be* that there is a difference in fixation duration-based metrics and saccade amplitude-based metrics. This prediction can be made regardless of any manipulations of independent variables in a study.

With that in mind, we advise that researchers consider how differences in trial durations (i.e., number of fixations) could influence the predictions that they generate for their studies in order to avoid drawing conclusions that are a mere by-product of coarse-to-fine behavior. In the context of Godwin et al. (2015), we dealt with this by comparing duration-based measures for trials that ended early (i.e., less than the median number of fixations) or late (i.e., higher than the median number of fixations) – at best, this was an approximate solution, and we would hope that the field will be able to develop a more complete solution in due course. Of the papers that we examined as part of our *Brief review*, the issue of coarse-to-fine behavior was relevant to their analyses in 80% of the sample. Of these, only one paper (6.25%) took coarse-to-fine behavior into account (Anderson & Humphreys, 2015). Coarse-to-fine behavior was not deliberately accounted for by the authors in this paper, however; instead, they took coarse-to-fine behavior into account by examining the first and second fixations made during search in separate analyses. It is possible, therefore, that many published findings in the existing literature could be undermined by this pitfall.

It is worth noting that although we identify coarse-to-fine behavior as a potential confound when comparing search trials that vary in their fixation counts, the variability in fixation counts and subsequent variability that appears in amplitude- and duration-based metrics can be a source of valuable information on a searcher’s criterion across trial types, as a shifting criterion will impact the diligence with which a participant engages and disengages with distractors. The important message is that, given the multitude of possible eye-movement metrics available to them, researchers should thoughtfully adopt a metric that pairs well with their hypothesis and also be aware of potential confounding influences on those metrics, just as is the case with other behavioral measures.

### Procedure and flow for a visual search and eye-tracking experiment

In the previous section, we provided two examples wherein the basic mechanisms that underlie eye-movement behavior can derail a researcher’s ability to generate the correct predictions for an experiment. In this section, we move onwards through the research cycle to discuss the procedure and flow of events for a visual search and eye-tracking experiment. Before that can begin, we must first discuss the considerations when choosing which eye-tracker to use for a study.

#### Choosing your eye-tracker

The choice of which eye-tracker to use determines more than simply the hardware that is employed, because there are a range of eye trackers available to purchase, and among them they provide a wide variety of options in terms of how eye movements are recorded, processed, and analyzed. Because the eyes move continuously, including making very small movements called microsaccades (for a review, see Martinez-Conde et al., 2013), improvements in the data quality are important for ensuring that recorded data provide as close to a veridical account of the true eye-movement behavior as possible. Unfortunately, attempts to reproduce the advertised precision of some eye trackers have not always been successful (for an overview of this, see Nyström et al., 2013).

Researchers have quantified eye-tracking data quality according to two metrics (for further discussion, see Holmqvist et al., 2011; Reingold, 2014). First, they have quantified data quality in terms of its accuracy (i.e., the distance between the veridical fixation point and where the eye tracker reports that fixation point to be) and precision (i.e., how reliably the eye-tracker reproduces a measurement of where the eye is fixating). As one might expect, accurately and precisely being able to pinpoint where the eye is directed in the environment is a vital component of any eye-tracker.

Beyond both of these metrics, the temporal precision of the tracker is also important in understanding how long fixations and saccades last. As the temporal precision decreases, the likelihood that a fixation or saccade will be “missed” increases. Saccades are typically very brief: In Godwin et al. (2015), saccades had a mean duration of 48.9 ms (*SD =* 23.56 ms). In a worst-case scenario, if a sample is taken from the eye before a brief saccade begins, then that brief saccade can be completed by the time the next sample is taken. Similarly, as the spatial precision decreases and/or the objects in the display decrease in size, we can begin to question whether or not some studies could reliably detect fixations upon those objects (for a more detailed simulation of this important issue, see Orquin & Holmqvist, 2018).

Given the considerations of eye-tracking data quality, which eye-tracker is the best choice for visual search and eye-movement experiments? The simple answer to this question is: “it depends.” It depends on the goals of the research being conducted, the size of the stimuli, their location on the display, and the ability of the eye-tracker in question to accurately localize fixations as landing on or around stimuli of the size being used in a given study. For example, if a researcher is interested in studying fixation durations but not locations, an eye-tracker that has higher temporal but lower spatial precision may be sufficient. Or to take another example, if the objects being examined on the displays are relatively large and spaced far apart, then an eye-tracker with lower spatial resolution may provide the same results as one with a higher spatial resolution. Consideration of this nature are especially important given that eye-trackers with the highest spatial and temporal precision can be very expensive. Yet, despite that, opting to use an eye-tracker that has been found to have lower data quality leaves a researcher open to the possibility that their findings will not be replicated when a tracker of a higher data quality is used, so caution must be exercised in this regard.

Within our sample of papers from *Attention, Perception, & Psychophysics*, the majority (85%) used Eyelink eye-trackers, and regardless of which eye-tracker was used, the majority of papers (85%) reported the temporal resolution used (some eye-trackers have fixed temporal resolutions; others do not). In Godwin et al. (2015), we did report the temporal resolution of the Eyelink 1000 that was used, recording at 1,000 Hz, i.e., one sample per millisecond. To date, there is evidence that Eyelink trackers have a high level of data quality (Ehinger et al., 2019; Holmqvist, 2017; Wang et al., 2017). Of course, other trackers are available for purchase and use, but again we would recommend above all else that prospective eye-movement researchers carefully examine the existing literature regarding data quality of different eye-trackers and consider the limitations of any tracker they might wish to purchase against the nature of the experiments that they plan to conduct. Given the popularity of Eyelink trackers in our *Brief review*, the remainder of this tutorial review will assume that the reader will be using an Eyelink tracker for their own research in the future. We do, however, believe that the review here and the points being made will apply more broadly to all studies of visual search and eye movements.

#### Prototypical visual search and eye-movements experiment

**Fig. 4:**
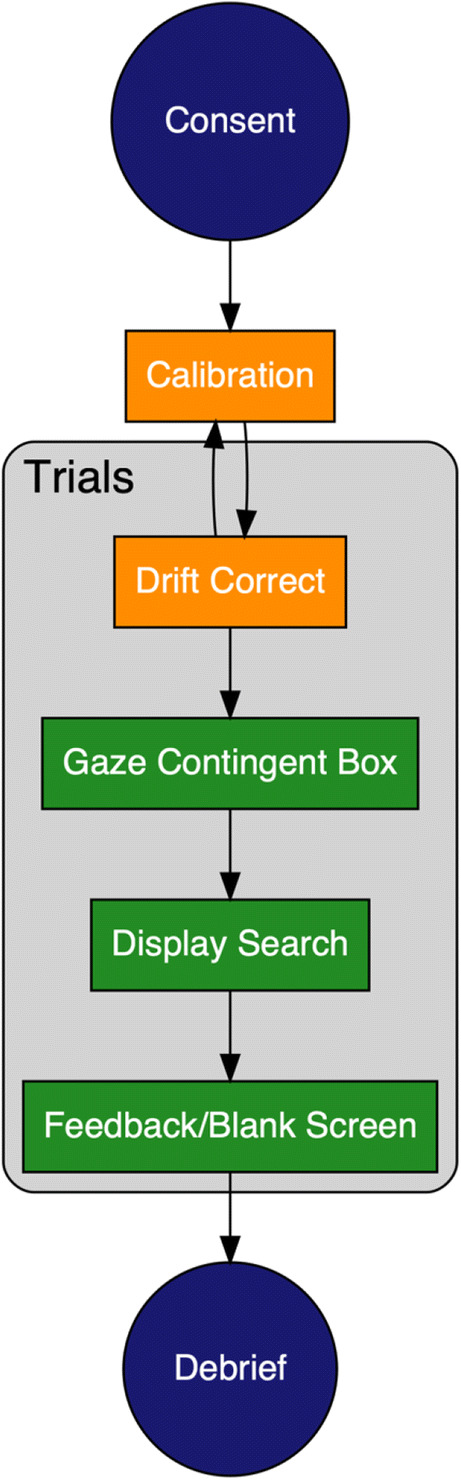
Procedure for a prototypical visual search and eye-movements experiment

We can now proceed to outlining a prototypical study of visual search and eye movements. The basic series of events during a prototypical visual search and eye-tracking experiment is presented in Fig. 4. Within our prototypical experiment, the process begins with informed consent from the participants. Participants are then typically seated in front of the eye-tracker and computer monitor, ready for the experiment to begin. We now walk through the process of setting up the eye-tracker and engaging in data collection.

##### Setting up and calibrating your eye-tracker

Some eye-trackers record data from both eyes, and others give the option to select one (or both) eyes to record. Similarly, some trackers permit free head movement during a study, and others require participants’ heads to be held relatively still through the use of a chin rest. In our *Brief review*, 40% of the papers reported recording from one eye only, and 35% reported which (eyes) were recorded from. In Godwin et al. (2015), we reported recording from the right eye only.

During the calibration procedure in Eyelink (and other) trackers, participants fixate a series of dots on the display in order to enable the tracker to more precisely and accurately determine the point of fixation. These calibration dots can be set up in default locations (e.g., there is a 9-pt calibration procedure), or can be placed in custom locations if desired. In the context of Godwin et al. (2015), we used (and reported using) a 9-point calibration procedure, which is ideal because the locations of the points span the far reaches of a display, providing reassurance that when the participants examine those far reaches, the reported eye position can be relied upon. For our *Brief review*, we found that only 65% of the sample gave information regarding their calibration procedure. Only 50% of the sample reported the number of calibration points used, and most of these (90%) used a 9-point calibration procedure.

After the initial calibration procedure in Eyelink trackers, a “validation” procedure begins, wherein participants are asked to fixate objects at the previously presented locations again, and the tracker measures the distance between the actual dot locations and where it computed the participant’s gaze to be. The error in these distances is presented for each point, along with maximum, minimum, and average error levels.^4^ Validation enables researchers to report these error values, and to set criteria for acceptable error in calibration; note that not all eye-tracking systems include this validation procedure. In Godwin et al. (2015), we reported that a calibration was accepted only when the average calibration error was below 0.5° of visual angle, with none of the 9 points exceeding an error level of 1° of visual angle. In our sample of papers drawn from *Attention, Perception, & Psychophysics*, only 30% reported the acceptance limits for their calibrations. Of these, there was a variety in different limits, which is to be expected given the range of stimuli and approaches used in different experiments (see OSM for further details).

Occasionally, a participant is not able to be calibrated in an eye-tracker, and/or their calibration falls below the required threshold. This can occur for a wide variety of reasons, which have been examined in a handful of studies. To take but a few examples, these reasons include the age of a participant, their eye color, whether they are wearing make-up, and whether they are wearing glasses (Dalrymple et al., 2018; Nyström et al., 2013; Wass et al., 2014). Ethnicity has also been found to influence the data quality when engaged in eye-tracking (Blignaut & Wium, 2014). Moreover, even after adopting a rigorous methodological protocol, it has been found that data quality can vary between different researchers running a given study (Hessels & Hooge, 2019). In a rather sobering observation, Holmqvist (2017, p. 23) notes that to obtain the highest data quality, “For most eye-trackers, it is best to recruit tall, male participants in the age around 17–25 years …It is easy to suspect that this description also fits many of the technical developers in the manufacturer companies, those who … repeatedly test, optimize and retest the prototype of the eye-tracker on themselves.”

Aside from the basic issues of calibrating participants, the manner in which calibrations are reported is quite variable. Some studies report the number of participants who are unable to be calibrated (25% of papers in our *Brief review*, and although we reported no participants having failed to calibrate in Godwin et al. (2015), it is possible that others were unable to be calibrated; poor long-term record-keeping does not permit a check in this case). Not all of the participants who begin a study complete it; other participants do not complete an eye-tracking study because calibration becomes difficult at a later point, perhaps due to fatigue, boredom, or other factors (zero papers reported in our *Brief review*; none reported by Godwin et al. 2015).

For those participants who can be successfully calibrated, due to a number of different factors – for example, moving, fidgeting, leaving the room for a bathroom break, operator error, computer error, or even taking a “selfie” in the eye-tracker^5^ – calibrations will sometimes need to be repeated during the course of a study. At the very least, a calibration will need to be repeated if the observer makes an overt movement away from the eye-tracker, such as standing up during a break or moving away from the tracker (if they had been resting their head in a chinrest). Studies will typically report a phrase along the lines of a calibration being repeated ‘after a break or when necessary’ (50% of the papers in our *Brief review* reported a note along these lines as part of their procedure). In Godwin et al. (2015), we repeated calibrations after a break of 50 trials, or when it was necessary.

The “when necessary” component here might sound rather subjective, but the act of calibrating a participant (as noted above) is somewhat of an art. Researchers balance the need for high-quality calibrations against the fact that repeatedly re-calibrating following even a small movement will eventually tire out the participant and cause them to be unable to complete the study. One way of double-checking calibrations without fully repeating the calibration procedure is via the use of the so-called *drift correct* procedure. During a drift correct, a single, central dot from the calibration process is presented to the participant, and they are asked to fixate it. If they are able to achieve a stable fixation on that point when asked, the next trial can begin. If the participant is not able to achieve a stable fixation point at the dot, or if, say, several trials in a row show evidence of the fixation point falling below the dot (indicating a downward “slip” in the calibration), the experimenter can pause the study and re-calibrate. We believe that this is typically what is meant by the “when necessary” clause in *Methods* sections of papers (it certainly is what we mean when we use such clauses in our own work). Aside from drift corrects, because the tracker usually displays the visual search display with the fixation point overlaid upon it to the researcher, the researcher can often quickly spot areas where the calibration is no longer accurate, such as when fixations all suddenly begin to land just under all objects, suggesting that there has been a slip in the calibration.

**Fig. 5:**
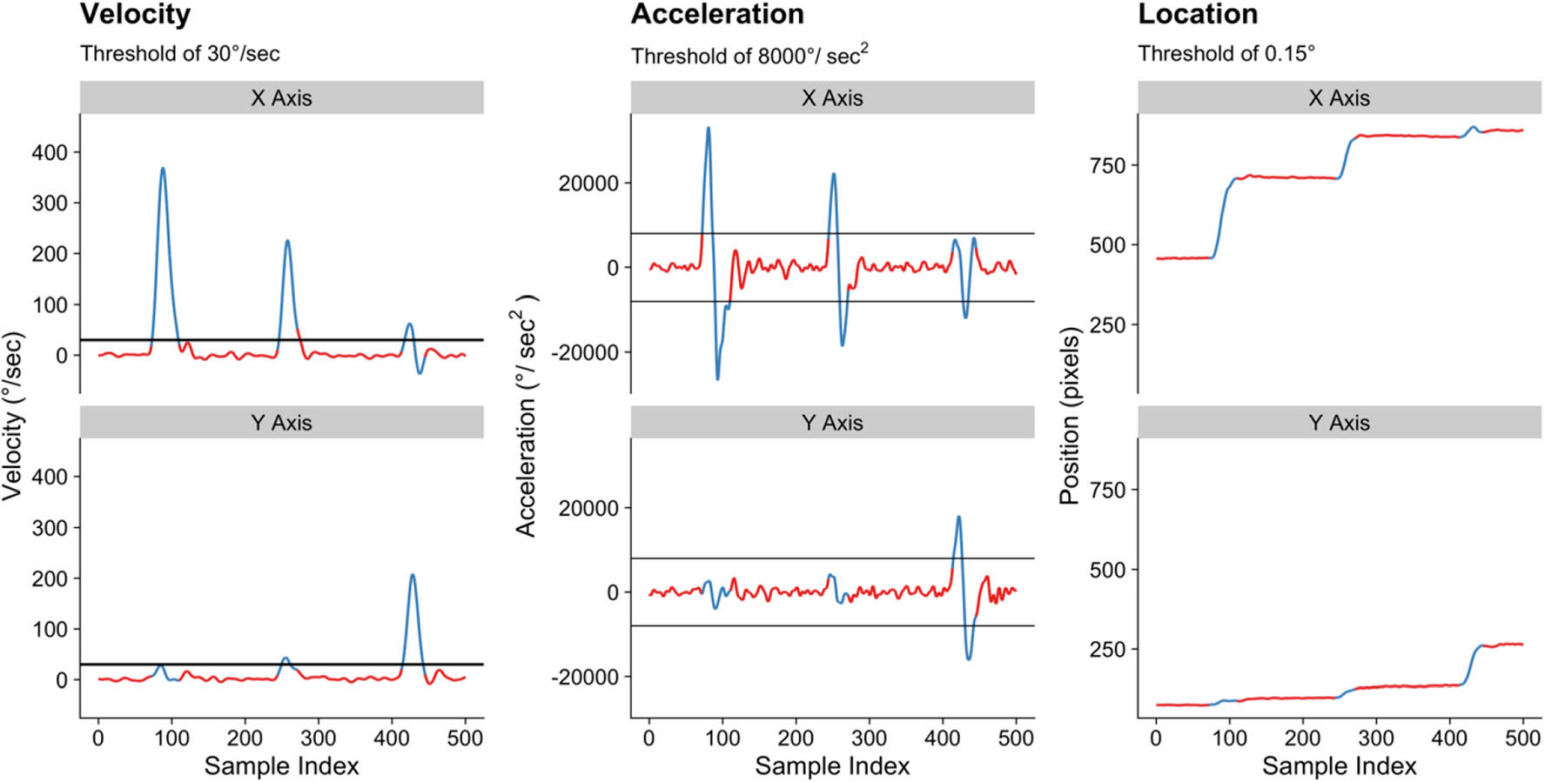
Three fixations and their surrounding saccades, broken down in terms of velocity (**left panel**), acceleration (**central panel**), and location (**right panel**). *Note.* Red lines denote samples computed by the tracker to involve a fixation, blue lines denote samples computed by the tracker to involve a saccade

There is one final detail to consider in relation to how an eye-tracker is set up before we can move forward: the algorithm used to detect fixations and saccades. As we noted above, the eyes are never still, and so an algorithm is required to interpret the raw information regarding the computed point of fixation in the environment and convert that into a meaningful measure of eye-movement behavior. In Fig. 5, we break down a series of fixations and saccades in a visual search task^6^ into the raw *samples* that they are based upon. Eyelink eye-trackers can be set in different ways to decide which of these samples are coded as a fixation and which are coded as a saccade. The algorithm used by Eyelink eye-trackers draws on three thresholds to determine whether the eye is making a saccade or a fixation (SR Research, 2017), which are denoted by the black horizontal lines in Fig. 5. When any of these thresholds is exceeded, the sample is coded as a saccade, otherwise the sample is coded as a fixation. The first of the thresholds is the velocity of the eye (see left panel of Fig. 5). The recommended defaults are to use a threshold of 30° of visual angle/s for visual search experiments wherein the stimuli do not move (different recommended defaults are available for other forms of experiment, such as reading research). The second of these is the acceleration, with a recommended default of 8,000° of visual angle/s^2^ (see central panel of Fig. 5). The final threshold is how far the eye moves between samples, with the default recommended to be 0.15° of visual angle (see right panel of Fig. 5, plotted in terms of raw X and Y display co-ordinates to make the overall movement of the eye across the display more easily recognized).

Within Godwin et al. (2015), we failed to report the saccade detection thresholds used in the Eyelink tracker but did in fact use the recommended defaults for experiments of this type (i.e., the same thresholds listed in the previous paragraph). Within our sample of papers from *Attention, Perception, & Psychophysics,* there was mixed reporting on the algorithms used when recording the data. Overall, 70% of the papers reported the algorithm used to parse fixations and saccades, but after that point these papers diverged from one another. Of that 70%, or 14 papers in our sample, there were a reported 11 different combinations of settings used to parse the fixations and saccades (see OSM for full information). For the sake of consistency, we would recommend researchers adopt the practice of using the recommended defaults for cognitive experiments for their given eye-tracker.

The discussion above demonstrates that at a most basic level, there is a lack of clarity and consistency within the field regarding the reporting of calibrations of eye-trackers during visual search experiments, the calibration quality, and the algorithms used to decide the most basic units of eye-movement behavior: what constitutes a fixation versus what constitutes a saccade. We have not labelled any of these issues as pitfalls because they do not in and of themselves result in proven or easily demonstrable errors in research – they are more a case of inconsistencies in the methods and/or reporting practices, and it is unclear as to what extent these inconsistencies affect the conclusions drawn from visual search and eye movements studies. However, despite that, it is worth noting that inconsistency and a lack of transparency have been argued to open up a field to widespread cases of Questionable Measurement Practices because researchers are able to obfuscate methodological and analytic decisions that can, in turn, “create” significant results that would otherwise not have been present in a dataset.

#### Recording eye-movement behavior during a visual search trial

By default, when using an Eyelink, the drift correction procedure involves asking participants to fixate upon a central point in the display. Once the drift correct procedure is complete, the search trial will typically begin. One might be forgiven for assuming that the lag between the drift correct ending and the search trial beginning will be zero – in other words, that the tracker will begin recording immediately after the initial drift correct disappears from view. Unfortunately, that is not the case. Instead, the procedure is as follows:

1. Drift correct accepted by researcher
2. Eye-tracker is turned on and recording starts (variable time required)
3. Search display appears (variable time required, depending on computer hardware, monitor refresh rate, etc.)

The time required for step (2) above is often longer than one might anticipate. In the context of the software and hardware setup used by Godwin et al. (2015), there is typically a delay of around 200 ms between the drift correct and the onset of recording. During that time, a participant can, for example, make a series of new saccades. One would hope, and likely assume, that the participant would keep their eyes focused on the center of the display until the search display appeared. This is perhaps an understandable assumption, because standard search tasks often involve trials being preceded by a fixation cross, so the drift correct procedure here is effectively replacing what is a standard in search experiments. But there is a problem in that the lag between the drift correct ending and the recording starting is long enough for participants to move their eyes away from the center point in the display, which brings us to our next pitfall.

#### Pitfall #3: Failing to ensure that participants begin by fixating the desired starting point of the search display

It is commonplace for researchers to record eye-movement behavior to better understand *guidance:* that is, the degree to which search can be guided towards specific objects. If, and only if, participants have the same starting point can any measure of guidance really be considered a fair comparison. If some participants “cut corners” and fixate an empty region that later contains an object (when the search array appears), researchers could then, without checking this issue, conclude that the participant wanted to fixate that object. Despite this, perhaps one of the most problematic assumptions in the study of visual search (and indeed, all of visual cognitive research) is that placing a fixation point at (typically) the center of a display will mean that participants, without a doubt, will be fixating at that point when asked to do so. This is simply not true, and that is for a number of reasons. First, our eyes are never still, and it can be uncomfortable holding a fixed gaze point for an extended period of time. The reader can try this for themselves by simply focusing on the full stop at the end of this sentence for a long period of time. Second, a consequence of using common participant pools like undergraduate university students is that participants are generally motivated to finish an experiment and leave the study as rapidly as possible. As a result, if they can find a route by which they can “cut corners” and move away from the center of the display (where no objects are typically placed) to a different location (where objects are likely to appear), then they will take that route as often as possible because they will be able to more quickly make a decision regarding the display presented to them and therefore complete the study sooner.

It could be argued that some of the points made in the previous paragraph are based on personal and subjective experience regarding participants being unwilling to maintain fixations at a specific point on the search display at the start of each trial. So let us turn to one study that we examined as part of our *Brief review* for further evidence to back up this point. This study (Ng et al., 2018) was not included in our *Brief review* because, despite using an eye-tracker during a visual search task, no eye-movement-based dependent variables were analyzed. However, it is the exclusions in this study that are of interest here, because one condition asked participants to maintain fixation at the center of the display during the trials. Across three experiments involving 122 participants, 34 were removed from the analyses for failing to maintain fixation on the center of the display (~28% of participants).^7^ It seems that participants simply are unable or unwilling to hold their fixation at (in this case) a central point for an appreciable period of time.

The solution to this pitfall of not ensuring search begins at the desired starting point in the display is to adopt the use of a “gaze-contingent” region that appears *after* the drift correct procedure. Gaze-contingent approaches involve changing, shifting, or updating the visual display contingent upon the position of the eyes. This gaze-contingent region in Godwin et al. (2015) was a reminder of the target images presented in the center of the display. Participants were asked to fixate this region and only after it had been fixated for a pre-set time did the search array appear. Because the gaze-contingent region was presented during the search trial itself, when the eye-tracker was already recording, the delay between the gaze-contingent region disappearing and the search display appearing was minimized. The result was that participants generally began each search display with their eyes aimed at a location close to the center of the display.

**Fig. 6:**
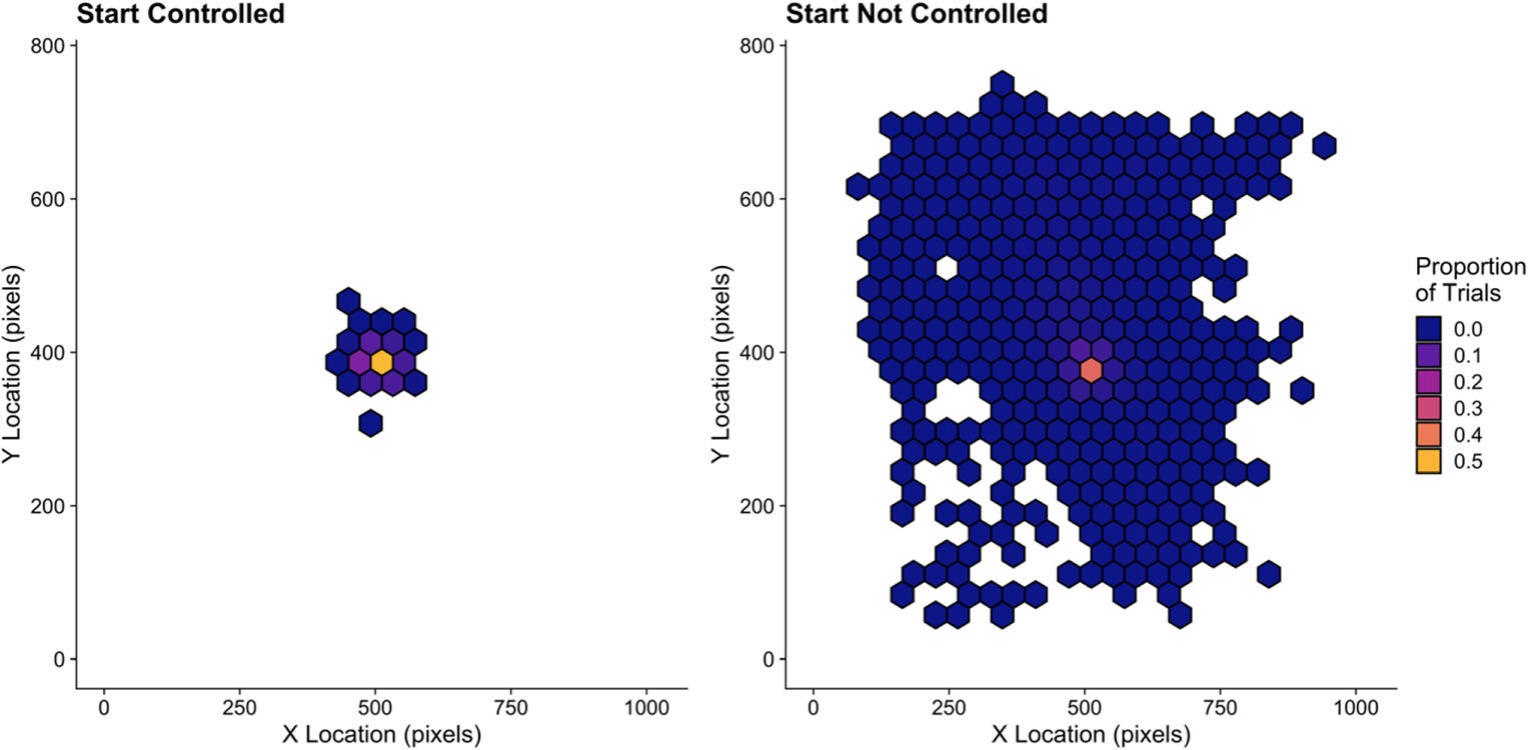
Starting locations when the start position is controlled (**left panel**) and when it was not controlled (**right panel**)

Indeed, we can quantify the effectiveness of the gaze-contingent approach by comparing the start locations for each trial in Godwin et al. (2015) with those from a separate set of experiments that did not use such a constraint (Godwin et al., 2013). In both sets of experiments, participants were supposed to begin their search in the center of the displays. Although there are a larger number of trials in Godwin et al. (2015) than Godwin et al. (2013) – around 20,000 versus around 11,000 – there is a stark difference in the overall pattern of start locations presented in Fig. 6, which can be seen in terms of the proportion of trials where participants are fixating each location when the search array appears. Clearly, it is vital to control the start locations of visual search to avoid this variability contaminating one’s data.

Within our *Brief review*, we found that 35% of papers took this issue into consideration and used the gaze-contingent approach described here. It also seems important to check that any such approach was successful. In our *Brief review*, just one of the papers (5%) did so. In Godwin et al. (2015), we must admit to failing to double-check fixation locations even using the gaze-contingent approach. Figure 6 makes it clear that there were a proportion of trials – albeit a small one – that began farther away from the center point than might have been hoped, *despite the use of the gaze-contingent approach*. Furthermore, in Godwin et al. (2015), 6% of trials involved a saccade away from the center being initiated within 80 ms – a duration so low that the saccade was likely planned and initiated before the display had changed, suggesting that it is very unlikely that this saccade was targeted based on visual information available at the time that it was planned (as would otherwise be assumed).

This pitfall is likely to have an *even worse impact* upon a study when participants can know in advance the locations where the objects will appear. It is common in studies of visual search and eye movements to engage participants in searches of circular arrays of objects (e.g., Findlay, 1997; McSorley & Findlay, 2001; Mestry et al., 2017), a technique used in 45% of papers in our *Brief review*. In these types of tasks, one might expect participants to very easily move, if they are not restricted, to fixate a location where an object will appear, even when it has not yet appeared. Caution is therefore required, if not at the point of data collection, but certainly at the point of data cleaning and processing, to ensure that participants behaved as expected before analyzing results. A tell-tale sign of problems here would be that participants fixated objects at an implausibly rapid speed, a fact that may be concealed by averaging across trials without checking the values individually before doing so.

The discussion in this section so far has covered most of what takes place in the repeating trials outlined in Fig. 4. In our prototypical example, after a participant has responded, the trial ends, and they are given feedback (auditory or visual) regarding whether or not they were correct in their response. This process continues in a loop until the trials are completed, the participant is debriefed, and the study ends.

### Cleaning your data

Up to this point, we have described pitfalls and issues relating to the setup and collection of eye-tracking data. We have outlined three pitfalls so far. By now, in our prototypical schematic search study outlined in Fig. 4, the data collection is complete and ready to be analyzed. How does this proceed, and what further pitfalls lie ahead? The data processing pipelines used to clean visual search and eye-movement experiments require multiple processing steps, along with decisions – both implicit and explicit – regarding how to clean, filter, and aggregate the data. The complexity of eye-movement datasets leaves researchers open to becoming lost in the so-called “garden of forking paths” when it comes to data-processing pipelines (Gelman & Loken, 2014). Given the pitfalls already described, the data-cleaning process has the potential to compound any pitfalls that have arisen during the setup and data-collection process.

Here, we describe several different sets of cleaning processes that can be applied to data processing pipelines in visual search and eye-movement experiments. Before doing so, it is worth noting the consequences of failing to appropriately clean one’s data. In a best-case scenario, including data that should not be included will add noise to the analyses. Optimistically, if that noise constitutes only a small fraction of the overall dataset (which is likely to number many tens of thousands of data points that *should* be included), then hopefully the noise should not change the outcome of any statistical tests carried out. Of course, that best-case scenario becomes increasingly unlikely as the different dimensions upon which data points should be removed stack up. And, as we have already seen, pitfalls can stack up very quickly indeed.

#### Fixation durations

Fixation durations can vary greatly. As noted above, it has been found that as the similarity between a given object and the target(s) being searched for increases, so too do the fixation durations. Setting appropriate thresholds to determine what values should be deemed outliers is, of course, an important step in ensuring accurate data cleaning protocols prior to beginning any analyses. Orquin and Holmqvist (2018) highlight a rather surprising example wherein the cutoff for fixation durations has been set inappropriately. Jansen et al. (2005), they note, set a cutoff of 300 ms such that fixations below this duration were excluded from any analyses. This value unfortunately included the median, leading to a very substantial exclusion rate of fixations. When it comes to fixation durations, it is important to note that a fixation’s duration can be classed as an outlier not just because the participant has engaged in an unusual behavior or made an error (as might be inferred for very long or very short RTs). Beyond that possibility, fixation durations can also be exceedingly long or exceedingly short due to a failure in the eye-tracking parsing algorithm. Very long fixation durations could be due to a failure to separate two fixations, with the tracker instead grouping them into one very long fixation. Similarly, brief fixations could be tracker errors wherein one fixation is erroneously parsed into two very brief fixations. This additional source of measurement error makes it all the more important to clean fixation durations.

Within Godwin et al. (2015), fixation durations of less than 60 ms or greater than 1,200 ms were removed as outliers. These bounds were not selected upon the basis of existing research or analyses suggesting that 60 ms and 1,200 ms are optimal for the removal of outliers; rather, the first author there and here chose them based on conventions within his laboratory, adopted from colleagues and collaborators from whom he learned how to engage in eye tracking studies. Within our review of papers published in *Attention, Perception, & Psychophysics,* we saw very limited adherence to consistent outlier removal, with only 15% of papers reporting the removal of fixations as outliers (one paper removed fixations 2.5 SDs from the mean, one removed fixations 3 SDs from the mean, and one removed fixations < 50 ms). It is therefore unclear as to what the importance of certain fixation duration outlier limits is upon visual search and eye-movement studies. However, it seems reasonable to have at least a lower bound for outlier removal to avoid including fixations that are so brief that no visual information could be acquired during their limited duration.

#### Fixation locations

Aside from removing fixations on the basis of their duration, we can also remove fixations on the basis of their location. An obvious starting point for a cleaning procedure along these lines would be to remove fixations that are recorded as falling outside the bounds of the display. On this point we must admit that in Godwin et al. (2015), we did not engage in such cleaning. Had we done so, we would have removed a further 0.06% of fixations. Of the papers examined in our *Brief review*, we found no evidence of researchers explicitly checking for this. In some cases, this issue may not have been relevant since fixations landing outside the display were filtered indirectly through other means (e.g., by focusing on fixations that fell on the target only, which was, of course, within the bounds of the display). For this tutorial, we felt it was important to take a strict approach to noting others’ strategies in order to understand what is typically reported by papers on visual search and eye movements. Still, this may be a useful metric to check for in future research.

#### Event contingencies

One often-overlooked aspect of data cleaning in eye-tracking and visual search experiments is the role that events and the subsequent contingences they introduce play in influencing eye-movement behavior. Under this approach, events can involve display changes, button press responses, and other aspects of the search task itself imposed by the nature of the study. These events have their own time course and duration, but so do individual fixations and saccades. For example, some fixations will coincide with display changes and/or button presses, and others will not. As we shall see, the events that take place during a fixation or saccade (what we here call the *event contingencies*) can have a powerful influence upon those fixations and saccades and it is therefore vital to map them out and be careful with regards to selecting and filtering datasets. This brings us to our next pitfall.

#### Pitfall #4. Failure to consider and clean eye-movement data based on event contingencies

**Fig. 7:**
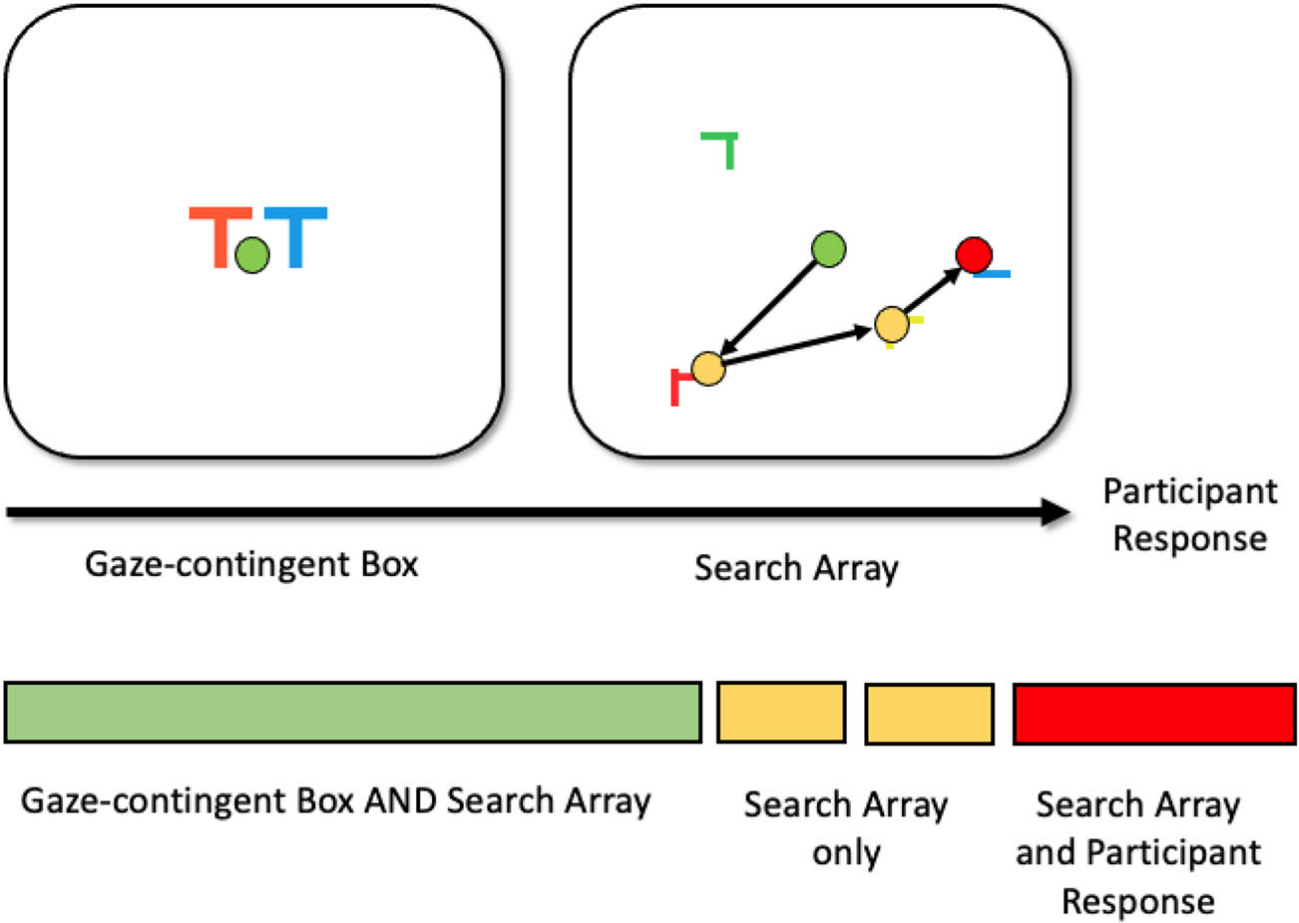
Overview of a search trial from Godwin et al. (2015)

To explain this pitfall, let us consider a simple search trial as depicted in Fig. 7. Here, a participant is presented with a drift correct, after which a gaze-contingent box is presented containing a preview/reminder of the target that is to be searched for. After the gaze-contingent box has been fixated for 500 ms, the search array appears. The search array remains visible until the participant responds.

The series of events that take place in a search trial can be regarded as *events,* and combining those events leads to a set of different event contingencies. Mapping out the different event contingencies is key in order to better understand and clean our data. If we take each event and combine them, as in Fig. 7, there are a distinct set of combinations of events that take place just in that one trial. There are fixations that:

- Began before the gaze-contingent box appeared and remained until the search array appeared (one fixation, light green color).
- Began and ended while the search array was visible (two fixations, yellow color).
- Begin when the search array was visible and involved a response from the participant (one fixation, red color).

These three different fixation contingencies are, we believe, uncontroversial and are entirely in line with what we might have expected to happen with participants following the nature of the task. But the problem is that these are not the *only* fixation contingencies that took place across the entire experiment, and it is worth mapping them out in detail. We do this in Table 1. As can be seen in Table 1, there was one trial wherein the participant began their fixation before the gaze-contingent box appeared, held that fixation while the box was visible, and still continued to hold that same fixation without moving their eyes before responding. Similarly, there was one trial where the fixation began while the gaze-contingent box was visible and that single fixation was held right up until a response was made. This is where the mapping out of event contingencies begins to be very helpful indeed: We would recommend removing these single-fixation trials because the participants did not, throughout the trials, move their eyes. Unfortunately, we did not remove these trials for this reason in Godwin et al. (2015), although one of the trials was removed because it resulted in an incorrect response.

**Table 1:** Fixation contingencies and their associated counts in Godwin et al. (2015)

Before gaze-contingent box	Gaze-contingent box	Search display	Response	Number of fixations
X	X			10,534
X	X	X		9,265
X	X	X	X	1
	X	X		10,738
	X	X	X	1
		X		127,312
		X	X	14,911
			X	11

The importance of mapping out event contingencies goes beyond merely screening out entire trials wherein participants did not engage with the task, however.

**Fig. 8:**
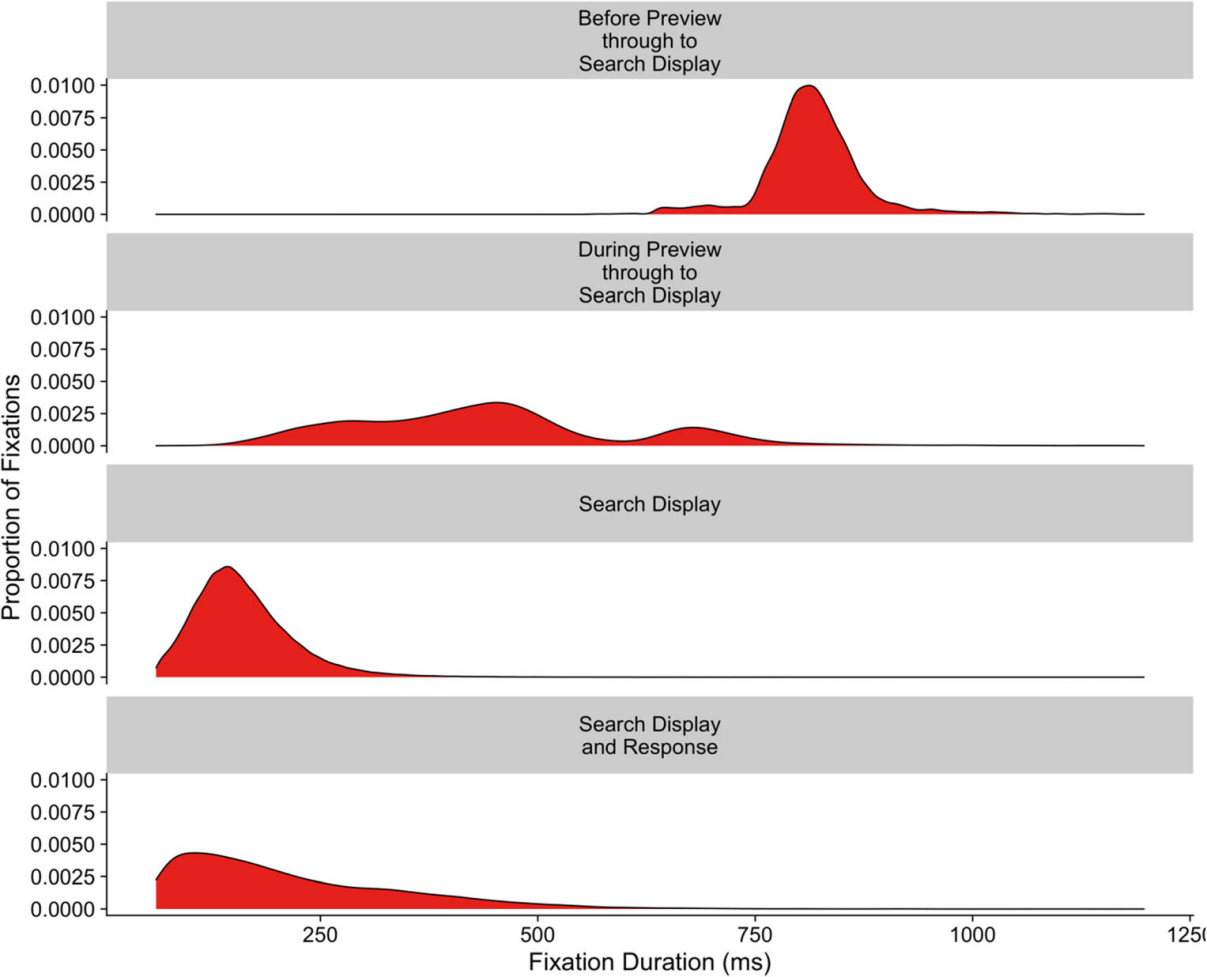
Density plots for the different fixation contingencies from Godwin et al. (2015)

Event contingencies are vital in removing fixations that should not be included in analyses. To highlight this, we can begin by turning to Fig. 8, which plots the distribution of fixation durations in terms of the different event contingences for Godwin et al. (2015). Here we have plotted only those contingencies that have a large number of occurrences.

The first row presents the distribution of fixation durations for those fixations that began before the gaze-contingent preview of the targets appeared, and then were maintained up to the point the search display appeared. These fixations were very long by necessity because they were maintained across the time period before the preview appeared, during the preview itself, and then ended after the search display appeared. The second row presents the fixations that began during the gaze-contingent preview and then persisted through to the onset of the search display. These have a multi-modal distribution, a pattern that is common for fixation durations when display changes occur, wherein fixations are often “paused” following a display change (e.g., see Henderson & Smith, 2009). They are therefore contaminated by the occurrence of a display change. The third row of Fig. 8 presents fixation durations for fixations that began and ended when the search display was visible. Their distribution has a gamma shape, a finding in common with studies of eye movements and reading (e.g., see Reichle et al., 2003). For any measures based on fixation durations, this will be the “cleanest” set of fixations to use since they are uncontaminated by display changes or button-press responses (we suspect that researchers often believe that they conducting their analyses upon this set of fixations when in fact, they are not). The fourth and final row presents fixation durations for fixations that occurred whilst the search display was visible but also coincided with a button-press response. As can be seen, these fixation durations have a different distribution compared to the rest, with a longer tail, suggesting that there is a fundamental qualitative difference between these and the fixations in the third row of Fig. 8.

The role that these event contingencies play is rarely recognized or discussed in visual search and eye-movement studies.^8^ We therefore would recommend researchers seek to be selective with regards to including or excluding fixations that coincide with display changes and/or button-press responses. The duration of these fixations can have unexpected properties and their inclusion could be problematic in terms of calculating appropriate values for statistical tests. In our *Brief review*, only one paper (5% of the sample) explicitly defined the time period under examination in each trial along with clear evidence of filtering by event contingencies. In Godwin et al. (2015), we did take event contingencies into account but were not explicit about the time periods under examination.^9^ One simple “rule of thumb” that could be adopted that we would recommend would be as follows: for time-based measures, we would recommend removing all fixations that coincided with display changes and analyzing them separately or not at all; for measures based on the probability of fixating objects, we would not remove these fixations since participants still did fixate the objects and this is an important detail to capture.

#### Landing positions upon objects

When searching, fixations will typically fall upon or near to the objects that are presented in each display. Landing positions of fixations have long been studied, and it has been found that fixations tend to land at the center of objects, and are normally distributed around that center point on both the x- and y-axes (Trukenbrod & Engbert, 2007). Other fixations sometimes land at the “center of gravity” between multiple objects (e.g., see Findlay, 1997; McGowan et al., 1998; Zelinsky et al., 1997), but this tendency is known to decrease as more objects are presented in a display (McSorley & Findlay, 2003).

As a consequence of fixation landing positions in visual search, different approaches have been taken by researchers when cleaning their data and assigning whether each fixation has landed upon an object. A strict approach would be to regard only those fixations that fell directly upon an object as having landed on that object. But doing so would likely be too conservative, given that undershoots and overshoots can occur, and also given the fact that the reported fixation position by the eye-tracker, as noted above, is the *average* location of the samples from that fixation, not the *only* location that the samples from that fixation landed upon. More importantly, a fixation falling *next to* a given object will likely still provide enough visual information about that object so as to enable identification of that item.

But how close does a fixation need to be to an object for it to count as being “next to” or “near enough to that object? The solution here is to choose the nearest object to the current fixation and treat the fixation as having landed upon that object so long as the fixation is within a certain distance threshold to the object. Here, the distance is expressed in terms as the distance from a fixation to the center of the nearest object.^10^ In the context of Godwin et al. (2015), a fixation was treated as landing on an object if it fell within 2.5° of visual angle of the center of that object. Of course, one could set an upper limit of effectively infinity here and treat *all* fixations as landing on the object that they were closest to. In our *Brief review*, we found evidence of different approaches taken by different researchers. Within our sample, the issue of the distance threshold to use was noted in 85% of the papers. Of these papers, 23.5% reported only selecting fixations that fell directly upon objects, 29.4% used a distance-based criterion, 11.7% reported treating fixations as landing on the object nearest to those fixations, 11.7% reported drawing a virtual box around objects, and 23.5% did not report their criterion.

We have not described the different approaches taken here by researchers as a pitfall, since it is unclear as to what the optimal approach actually should be. Certainly, upon reflection, one issue with the cutoffs used both in Godwin et al. 2015 and our *Brief review* is that justifications as to *why* a given distance cutoff was used are lacking. At the risk of being overly honest, in Godwin et al. (2015), the value for the distance threshold was chosen because it seemed a reasonable one to choose based on prior experience and previous papers. We suspect that this approach is not out of the ordinary for the field.

Given the (potentially) arbitrary nature of the thresholds chosen for determining whether a given fixation landed on a particular object, in some cases researchers opt for analyzing the dataset twice: once, using a stricter criterion of treating fixations as landing upon an object only if they were upon that object, and a second time using a more liberal criterion based upon the distance to the center of that object. In Godwin et al. (2015) we did not do this, but the first author here has done so elsewhere (Menneer et al., 2012). In our *Brief review*, we saw no evidence of researchers taking this approach. We return to the issue of which distances to use, with a potential solution, in the *General discussion*.

#### Incorrect responses

The final method used to clean eye-movement data in visual search studies is the removal of incorrect responses. Because it is unknown exactly why an incorrect response is given (on any particular trial) and whether it is the result of a lapse in attention, a mistaken button-press, or some other factor, visual search researchers typically remove incorrect responses from the computation of several metrics. This is described in more detail in the next section. We followed this convention in Godwin et al. (2015) and in our *Brief review*, 80% of the sample reported doing the same. Of all the different ways to clean data, this appears to be the most agreed upon of all.

#### Summary

In the present section, we have outlined a number of different routes that researchers can take to clean their data derived from visual search and eye-movement experiments. We have discussed outlier removal upon the basis of fixation durations, fixation locations, a consideration of the importance of event contingencies, landing positions upon objects, and incorrect responses. By this point it should be clear that this variety of options can create only one outcome: a variety of different approaches based upon the experience and understanding of a given researcher. These different approaches, along with the intrinsically subjective decision-making that can take place, serve to create an almost dizzying array of options. When combined with the pitfalls noted in previous sections, one can see how quickly even one error or pitfall that might have a minor impact upon the data can rapidly combine with a host of others to create a dangerous “cocktail” of pitfalls plagued with noise, methodological confounds, and theoretical flaws.

### Analyses

We have now reached the stage in our research cycle where analyses are conducted. Before delving into the details of the dependent variables examined during visual search and eye-movement studies, we begin by describing how those dependent variables are calculated, providing an overview on the different analytic approaches taken by researchers.

#### Analytic approaches taken in visual search and eye-movement studies

For the most part, in our *Brief review*, researchers primarily used Null Hypothesis Significance Testing to analyze their datasets (90% of the sample, with 15% using Bayesian analyses, and some using both approaches). In Godwin et al. (2015), we also used Null Hypothesis Significance Testing, focusing on ANOVAs and *t*-tests. Thanks to cross-pollination with psycholinguistics research, in the study of eye movements and reading it is now standard practice to use Linear Mixed Effects (LME) models rather than ANOVAs and *t*-tests to examine datasets (e.g., see Liversedge et al., 2016). Psycholinguistics adopted LME models to address the *language as fixed-effects fallacy,* the faulty assumption that a stimulus word list automatically generalizes to the population of words from which it was sampled (Clark, 1973). LME models increase statistical power and generalizability by including random effects associated with stimuli and participants in one equation. This practice does not seem to have transferred over to the study of visual search and eye movements, but we believe it could also be adopted as the current best practice in the analysis of visual search data. LME models offer a number of advantages over ANOVAs, including increased statistical power, increased recognition of individual variation between participants, and an ability to account for the effects that different stimuli have (Baayen et al., 2008). In our *Brief review*, we found only one paper (5% of the sample) using these models.

#### Pitfall #5. Failing to calculate appropriate values for each participant

In a standard visual search experiment that does not involve the recording of eye-movement behavior, calculating values such as the mean RT for each participant is a straightforward process. All that is required is to take the mean of the RTs from all trials where a correct response was made (we call these the *by-trial* RTs). The resultant *by-participant* means can then be entered into statistical tests such as ANOVAs and *t*-tests. Doing so is regarded as acceptable because (broadly speaking) the participants were usually engaged in the same number of trials and therefore this approach permits a fair comparison of the means between participants and conditions.

A very different situation arises when it comes to computing the averages for visual search and eye-movement studies. Because the number of fixations made per trial varies quite substantially (between 1 and 38, with a mean of 6.91 in Godwin et al. 2015), an additional step is required when computing the by-participant means. Suppose that we want to examine the mean fixation duration. We begin by calculating the average fixation duration per trial (the by-trial means). Then, we average these by-trial means, as with the RTs described in the previous paragraph, to create the by-participant means.

**Fig. 9:**
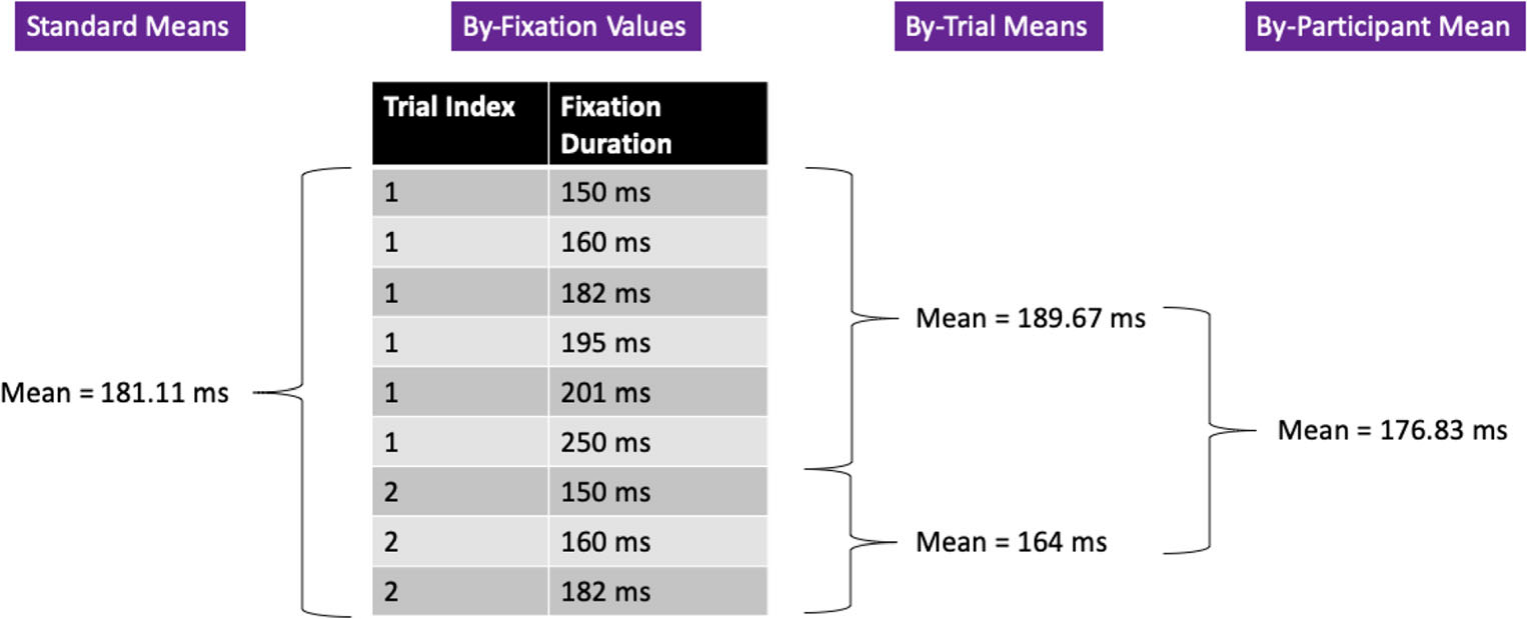
Two methods for computing fixation durations. *Note.* Because of course-to-fine behavior, the standard method for calculating by-participant means (seen on the left side of the figure) may lead to inflated values relative to a technique that constructs by-participant means from by-trial means (seen on the right side of the figure)

Why is the additional step required? Well, because the number of fixations made per trial varies substantially, if we simply skip the first step and take the average of *all fixation durations* to determine the by-participant means, then those by-participant means will be heavily biased by trials wherein there were a large number of fixations. We know already that coarse-to-fine behavior leads fixations to increase in duration as a trial progresses (as described above as part of pitfall #2), so the direct consequence of skipping the first step here will be to create exaggerated estimates of mean fixation durations. We sketch out this issue with an example in Fig. 9, where the means for two trials are computed. One trial has six fixations; the other has three. The durations of the first three fixations in both trials are the same. Without computing by-trial means first, the mean fixation duration is computed as 181.11 ms. By computing by-trial means before computing the final mean for this participant, the mean fixation duration is computed as 176.83 ms.

In Godwin et al. (2015), we made sure to compute the by-trial means first where appropriate, yet did not explicitly state this processing step in the manuscript itself. In our *Brief review*, one paper in the sample did not need to take this approach (because LME models were used and so analyzed the by-fixation values), yet for the rest this approach would have been potentially useful. Unfortunately, none of the remaining sample reported taking by-trial means into account when calculating final values for statistical tests. Given that the computation of by-trial means was not noted either by Godwin et al. (2015) or in our sample for our *Brief review*, it remains possible that researchers are cognizant of this issue, but are just not discussing it. That being said, we feel that this is unlikely since one might expect at least some mention of by-trial means within our *Brief review*. Moreover, of the authors of this tutorial review who regularly engage in visual search research, only one (HG) uses the by-trial approach, whilst the others (MH, SW) report having used the standard approach. The standard approach for authors MH and SW arises by the use of pivot tables in Microsoft Excel to rapidly compute by-participant means on large columns of values. Overall, then, we believe it is likely that researchers may be slightly inflating the raw values of any time-based measures when calculating means in the current literature. We suggest that researchers should be more explicit about the preprocessing stages they use to arrive at by-participant means, in service of enhancing reproducibility within the discipline.

#### Multiple comparisons

As we shall discuss in more detail in the next section, there are a very large number of different measures that researchers can use when analyzing data from eye-movement experiments. Some have argued that researchers should correct for multiple tests after running several statistical tests on different dependent variables in this manner (von der Malsburg & Angele, 2017). However, the argument against doing so is that many measures are related to one another, and so a correction for multiple comparisons does not need to be applied. For example, if one were to examine the total fixation duration (i.e., the sum of all fixation durations), and the number of fixations made per trial, one can be divided by the other to give the measure of the average fixation duration per trial. In Godwin et al. (2015), we did not utilize any corrections for multiple comparisons across different dependent variables, and this was consistent with the papers examined as part of our *Brief review* (0% of the sample corrected across different dependent variables). It is therefore likely generally the case that multiple correction penalties are not applied when researchers examine multiple DVs in visual search and eye movements experiments. Using such corrections, or using MANOVAs, for example, could account for this multicollinearity.

#### Summary: Analyses

In this section, we have discussed the different analytic approaches taken by researchers studying visual search and eye-movement experiments and highlighted one potential pitfall in terms of how mean values are calculated.

### Measures

At this stage in our walkthrough, we have reached the different dependent variables that can be examined as part of visual search and eye-movement experiments. We noted above that avoiding issues of multiple comparisons can cause concerns about the conclusions drawn from the statistical analyses in a study. However, when conducting confirmatory analyses above all else, it is of course vital that whatever measures are used to examine a dataset have a clear theoretical motivation.

We now proceed to discuss the different dependent variables that can be examined as part of visual search and eye-movement experiments in turn. We have divided the dependent variables into three categories. The first are *behavioral* measures. These involve measures derived from the button-press responses made by participants. The remaining two categories are both derived from eye-movement behavior and involve *global* eye-movement measures and *local* eye-movement measures. Global measures include all eye movements made during a trial period, whereas local measures break the data down and focus upon a set of objects or events in each search trial. For example, as a preview, global fixation durations relate to the mean fixation durations of all fixations in a trial whereas local fixation durations relate to the mean fixation durations when fixations fell upon specific objects (such as the target) in a trial.

#### Behavioral measures: Response accuracy and response times

Perhaps the most standard measures are those relating to behavioral responses made on each trial. These measures have some typical characteristics that are worth briefly noting. Both response accuracy and RTs were analyzed as part of Godwin et al. (2015), and 75% of the sample in our *Brief review* reported both of these measures.

Response accuracy was reported by 75% of the sample from our *Brief review*. In Godwin et al. (2015), we found that the higher-prevalence target was more likely to be found (i.e., had higher response accuracy) than the lower prevalence target. When response accuracy is not analyzed by researchers, it is typically because the task was very easy (i.e., targets are readily found and not often confused with distractors). One often-overlooked point is that response accuracy is generally higher in target-absent trials than target-present trials. It is believed that this is because even though a standard search study might present a target in 50% of trials, the probability that each individual object will be the target is quite small. For example, if a target is presented in 50% of trials, and there are 16 objects presented per trial, then, on average, the probability that any given object will be the target is 1/(16*2) = 0.03 (Godwin et al., 2017).

Response times are generally analyzed by researchers, including by Godwin et al. (2015), and by 95% of the papers in our *Brief review*. The standard finding is that RTs in correct-response target-absent trials are longer than those of correct-response target-present trials because more time is required to examine (almost) all of the objects in each display, and because search can be terminated when a target is found (Chun & Wolfe, 1996). In Godwin et al. (2015), for example, we found that participants were faster to detect the higher-prevalence target than the lower-prevalence target.

#### Global measures

**Fig. 10:**
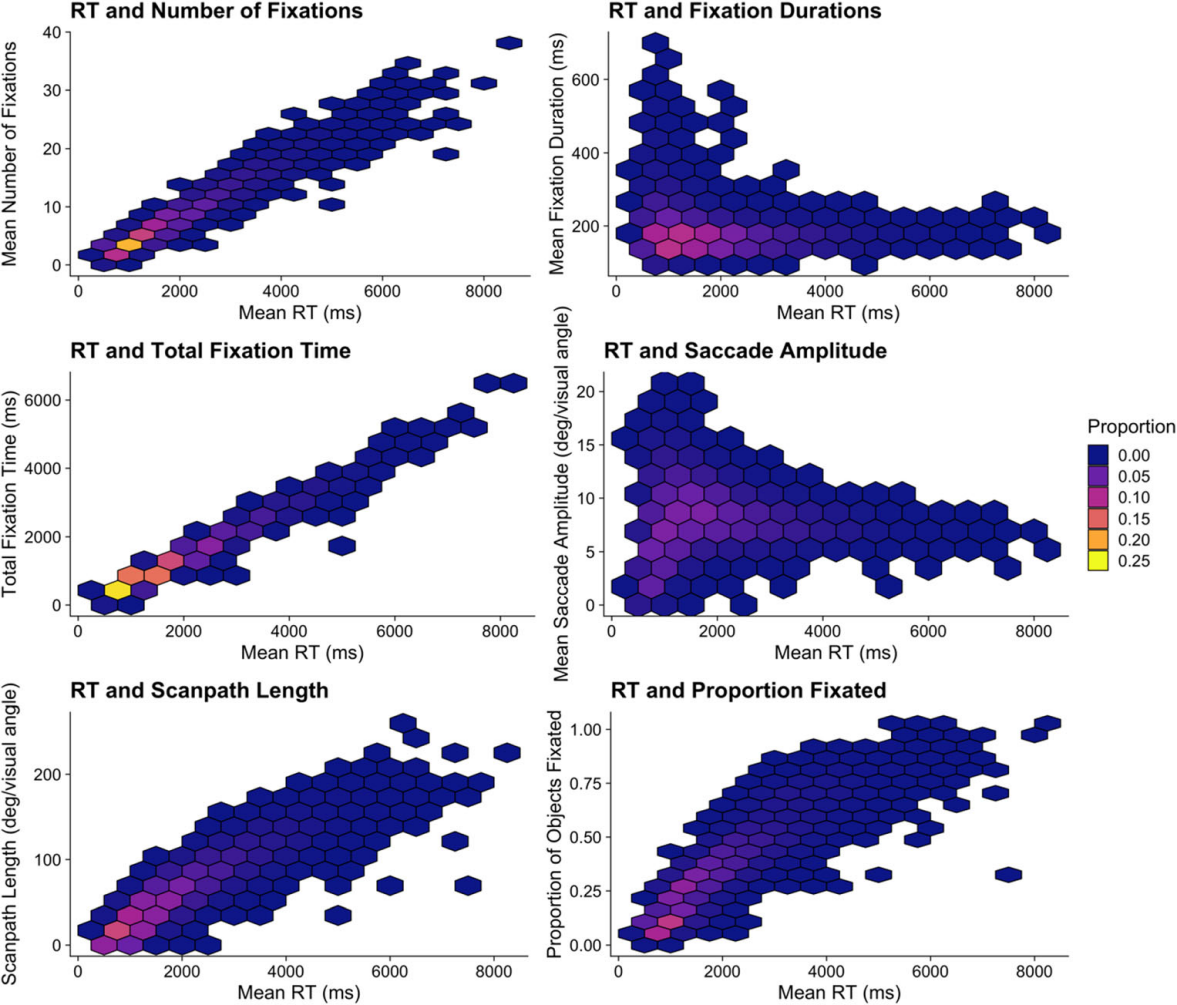
Heatmaps comparing mean reaction times (RTs) and a series of eye-movement measures from Godwin et al. (2015)

We next turn to discussing measures relating to eye-movement behavior. The first set of measures that will be discussed will be global measures. As noted above, these measures incorporate all fixations within a given trial period before means are computed. As will become clear, when putting the different measures together, a complex and highly correlated pattern emerges. We have touched on this issue when describing these measures in turn but plot out a number of relevant aspects of how they relate to one another in Fig. 10.

##### Fixation durations

Fixation durations are often used to examine the underlying information processing that takes place during visual search. As noted above, fixation durations are a clear reflection of the depth or detail of information processing during visual search. In Godwin et al. (2015), we did not examine global fixation durations; in our survey, 15% of the sample used this measure.

##### Total fixation time

The total fixation time is sometimes used to gain a measure of how long searchers spend searching the display (it is sometimes called total gaze time or total dwell time). It is simply the sum of all fixation durations per trial. As one might expect, total time increases when: (a) fixation durations increase (e.g., because search becomes more difficult), and/or (b) the number of fixations increase. Because total fixation time does not include saccade durations in its calculations, this measure is, by definition, shorter in duration than RTs (see Fig. 10). However, given its similarity to RTs, it is rarely reported in studies of visual search and eye movements because it provides little additional information beyond RT measures. In Godwin et al. (2015), we did not examine total fixation time at the global level; in the sample of papers examined for our *Brief review*, 0% of the sample used global total fixation time, suggesting this is not a particularly popular measure to use.

##### Number of fixations

The number of fixations per trial tends to increase as there are either more objects to examine, or because the search becomes more difficult. Much like total fixation time, it will generally be correlated with RTs because one would expect more fixations to be made as RTs increase (see Fig. 10). However, this might not always be the case. For example, RTs could increase simply because participants made more fixations of the same duration or made fewer fixations of longer durations. The distinction between these two different outcomes can sometimes be useful to researchers trying to better understand the shift in information processing and search behavior between different conditions. In Godwin et al. (2015), we did not examine the number of fixations at the global level; in the sample of papers examined for our *Brief review*, 25% of the papers used this measure, so, as with the other global measures of eye-movement behavior, this is not a particularly popular measure to use.

##### Saccade amplitudes

The length of a saccade that occurs between two fixations is referred to as the saccade amplitude, and it is measured in terms of degrees of visual angle. Although a less popular measure of search behavior, saccade amplitudes can be useful in providing an understanding of the extent or spread of searching a given display. Mean saccade amplitudes have a weaker relationship to overall trial RT than the number of fixations or the total fixation time (see Fig. 10). This is because the mean saccade amplitude does not ‘add up’ the different saccades made during a trial. In Godwin et al. (2015), we did not examine saccade amplitudes, and in our *Brief review*, only 10% of the sample used this measure.

##### Scanpath length

The scanpath length is defined as the sum of all saccade amplitudes in a given trial. The scanpath length can, rather like the saccade amplitudes, be used to gain a measure of the extent of searching a display, or the degree to which search is exhaustive – i.e., the degree to which participants search across the whole display. As can be seen in Fig. 10, the scanpath length is related to mean RTs in quite a clear manner. Longer trials are associated with longer scanpaths. We did not examine scanpath length in Godwin et al. (2015), and it was not used in any of the papers that we examined for our *Brief review*.

##### Proportion of objects fixated

The proportion of objects fixated provides a more direct measure of the exhaustiveness of search than does scanpath length, saccade amplitudes, or the number of fixations. As one might expect, increasing RTs will likely be correlated with participants fixating more objects (as can be seen in Fig. 10), but this may not always be the case. Instead, increases in RT may signify that the same number of objects are being fixated but instead participants are spending more time on each object. Indeed, in Fig. 10, the somewhat linear relationship for briefer RTs “flattens out” as RTs become longer, most likely due to a ceiling effect wherein there are progressively fewer “new” objects that have been left unvisited during the search. We did not use this measure in Godwin et al. (2015), and none of the sample for our *Brief review* used this measure either.

##### Proportion of objects revisited

**Fig. 11:**
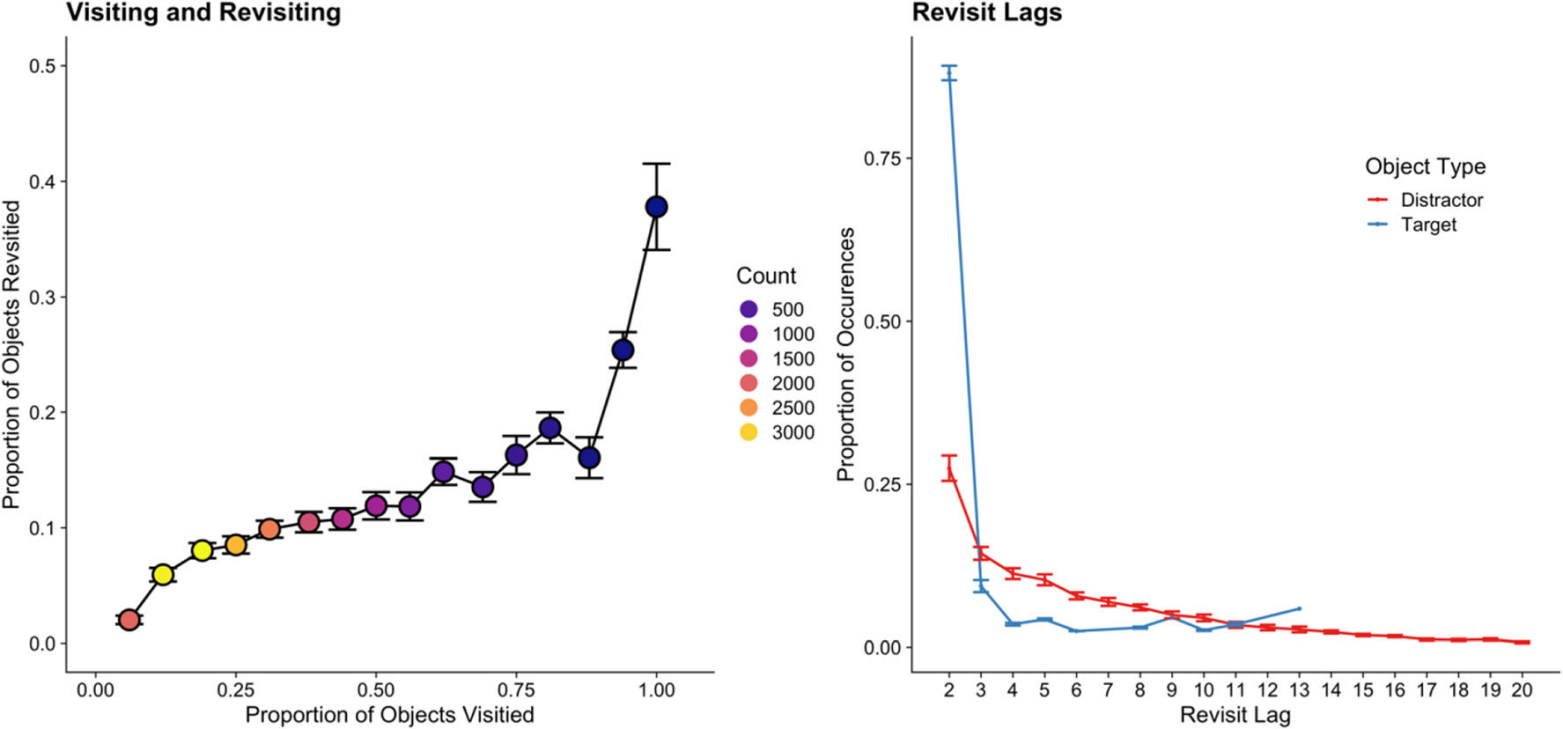
**Left panel:** Plot comparing the proportion of objects fixated with the proportion of objects revisited during visual search trials in Godwin et al. (2015). **Right panel:** Proportion of objects that were revisited as a function of their object type (distractor/target) and their revisit lag. *Note:* For the left panel, “Count” refers to the number of trials falling into each category. For the right panel, we calculated the values on the basis of objects that were only ever fixated twice, and excluded others. We also filtered the data to comprise objects that had a revisit lag of 21 or fewer

Revisits are thought to occur either because a searcher’s memory record for objects that have already been inspected has become exhausted (Peterson et al., 2001) or because a searcher returns to an object to check that object a second time, having fixated it too briefly on the first visit (Godwin, Reichle, & Menneer, 2017; Peterson et al., 2007). Based on this, we can expect the proportion of objects revisited to increase as the proportion of objects visited also increases (and this is shown for the data from Godwin et al., 2015, in Fig. 11). The proportion of objects revisited is sometimes used as a measure of how confident participants are in their search, with evidence that revisits become more likely, for example, when searchers are inexperienced (Godwin et al., 2015) or when searchers expect a target to appear (Godwin, Menneer, Cave, et al., 2015). In Godwin et al. (2015), we did not use this measure, and none of the papers examined as part of our *Brief review* used this measure.

##### Global measures summary

Throughout this section, we have described a series of different global measures of eye-movement behavior. These measures, whilst perhaps less popular than the local measures discussed in the next section, can nonetheless be useful in gaining theoretical insights into the overall pattern, extent, or spread of a visual search.

#### Local measures

To reiterate, local eye-movement measures are based upon a subset of events that take place in a trial, or a subset of the objects in each trial. The fact that they are based on a subset of the data means that they are often highly focused in order to answer a specific research question. Many global measures have equivalent local measures – a fact that we begin by describing in detail below.

##### Local measures of fixation durations, total fixation durations, number of fixations, probability of fixating, and probability of revisiting

We have already discussed global measures relating to fixation durations, total fixation durations, number of fixations, the proportion of objects fixated, and the proportion of objects revisited. Their local counterparts can be used in different ways, focusing upon a given object, class of objects, or events that take place during a trial (in our *Brief review*, 20% of the sample used local fixation durations, 30% of the sample used local total time measures, 50% used measures relating to whether or not objects were fixated, and 30% used measures relating to the likelihood or number of revisits). For example, a researcher might want to examine the total fixation duration on target objects and compare that with the total fixation duration on distractors (e.g., see Peltier & Becker, 2016) or compare one form of distractor to another (e.g., Hout et al., 2017). Elsewhere, researchers might want to compare the average fixation duration in one period of a trial with that of a different period (e.g., see Godwin et al., 2013).

When computing these local measures, an additional step is required to calculate the mean values. For some measures, such as the average fixation duration, a researcher will need to first compute a *by-object* value before computing the by-trial and by-participant means. This is for much the same set of reasons as described with regards to pitfall #5: It is important to ensure that all fixated objects have an equal representation when computing the means, just as it is important that all trials have an equal representation when computing the means.

Beyond this, when selecting a local measure, researchers need to carefully consider potential confounding factors to avoid engaging in Questionable Measurement Practices. These confounding factors can often involve other events that take place within a trial. For example, Orquin and Holmqvist (2018) recommend against the use of total fixation time as a local measure of the time taken to examine a given object. The problem, they note, is that objects are often visited more than once. The total time fixating an object could therefore comprise a series of fixations during a single visit upon that object, one long fixation upon that object, a few brief fixations on that object early on in a trial followed by further fixations later in the trial, and so on. The pitfall here is that researchers are casting too wide a net when operationalizing their dependent variables, which is resulting in potentially incorrect or confounded analyses. There are two potential solutions to an issue of this nature.

One solution is to not just rest all of the predictions and theoretical interpretations upon one single measure. Instead, researchers can produce a combination of predictions for different measures based upon their theoretical questions. Doing so can help to protect against a single, potentially imperfect measure being confounded and can provide converging evidence in support of a set of predictions. In Godwin et al., (2015), we were interested in determining whether low-prevalence targets were missed because they were not examined by searchers or whether they were missed after being examined. Addressing this question necessarily involves multiple measures, some based on the probability of fixating a target and others based on the probability of identifying those targets that were fixated. There were other measures besides these that were assessed but the point here is that often the only way to address a research question is to do so using multiple different measures. Overall, then, examining multiple measures can enable a researcher to gain a “bigger picture” of the results than just focusing on one measure.

A second solution is to make far more explicit and detailed predictions about local measures. If we turn to the study of reading and eye movements for inspiration and an example, it is commonplace to use a set of highly specific local measures when analyzing datasets. These include, for example, *single fixation duration*. That is, the duration of a fixation when a word is fixated only once. Such a measure is useful since it is a ‘pure’ measure of the time taken to fixate and fully process a word. Single fixation durations are typically longer than the *first fixation duration* on a word that is visited more than once because often the first fixation duration was erroneously too brief, leading to a need to revisit that word (for a review, see Rayner, 2009). We shall return to this issue later in the *General discussion*.

##### Time to initiate first saccade

In the *Data-cleaning section*, we discussed at length some of the issues associated with the first fixation position (and duration) in a visual search task. We noted that researchers often unintentionally fail to be certain that the starting fixation location is where it is assumed to be. Moreover, we discussed the varying factors that influence fixation durations for these first fixations that span the pre-search-display time period and the appearance of the search display. Putting those concerns to one side for a moment, we can discuss the time to initiate the first saccade. This measure is commonly used to assess the speed at which a searcher can localize and move their eyes to an object or area of interest within the display. It was often used in early studies of eye movements and visual search (e.g., see McSorley & Findlay, 2001, 2003); we did not use it as part of the analyses conducted in Godwin et al. (2015), but it was used by 25% of the papers in our *Brief review*. Given the potential issues with where participants begin a search, it is possible that, unless properly cleaned, existing research that has used this measure may need to be revisited.

##### Time to fixate objects

The time to fixate objects can be used as a global measure to assess the time taken to first fixate any object, but it is often more specifically used in a local, by-object fashion, examining the time taken from the onset of the search display to first fixate a given object. In most cases, the object in question is the target object. In doing so, researchers can then use the time to fixate the target as a measure of the efficiency by which the target can be selected from the background of distractors (Cain et al., 2013; Nodine & Kundel, 1987). The time to fixate the target increases as the target becomes more similar to other objects in the display, and as the number of target-similar objects in the display increases. In Godwin et al. (2015), we found that when the targets were dissimilar to each other, participants fixated the higher-prevalence target more rapidly than the lower-prevalence target. We took this to signify that higher-prevalence targets were more highly prioritized for fixations than lower-prevalence targets (see also Hout et al., 2015). In our *Brief review*, 30% of the sample used this measure.

##### Probability of fixating and then identifying

The probability of fixating and then identifying is a measure restricted to target objects only. It was first developed in the study of radiology and visual search (e.g., see Nodine & Kundel, 1987), inspired by the importance and interest relating to finding targets. Put simply, researchers wanted to better understand how and why targets were missed, and found that a substantial proportion of tumors were fixated but still missed by searchers. Generally speaking, participants are less likely to identify the target after fixating it when search becomes more difficult. In Godwin et al. (2015), we used this measure to better understand why low-prevalence targets are missed, and found that low-prevalence targets were more likely to be missed after being fixated than higher-prevalence targets (see also Hout et al., 2015). In our *Brief review*, however, no papers in the sample used this measure.

##### Verification time

Verification time is defined as the time between first fixating the target object and correctly responding that it is present (e.g., see Malcolm & Henderson, 2009). It serves as a useful complementary measure to the probability of fixating and then identifying a target because even if the target is found, the time taken to do so can increase when search becomes more difficult. In Godwin et al. (2015), we broke verification times up into two categories: early (faster than the median RT) and late (slower than the median RT). We did this because we expected lower-prevalence targets to be fixated later in trials. Given how coarse-to-fine behavior influences fixation durations, the concern was that because fixation durations increase as a trial progresses, the lower-prevalence targets, fixated later in trials, would be guaranteed to exhibit longer verification times than the higher-prevalence targets. In our *Brief review*, 40% of the sample used this measure in some shape or form.

##### Local measures summary

In this section, we have given an overview of some of the local measures that can be used by researchers when analyzing data from a visual search and eye movements experiment. As we have discussed, the primary challenge facing a researcher when using any local measure – be that in a visual search study or any other eye-movement study – is filtering the data in a manner that avoids as many confounds as possible. Nevertheless, local measures, by their more detailed and focused nature, are both more popular and more useful to researchers than most global measures because they enable researchers to answer more specific and more detailed theoretical questions.

#### Putting it together: Drawing conclusions about multiple different measures

As the preceding discussion and walkthrough has highlighted, there are a range of different measures that researchers can draw upon to analyze behavior from visual search and eye-movement experiments. On the one hand, researchers have argued against analyzing large numbers of measures in a single study in order to avoid false positives in statistical tests (von der Malsburg & Angele, 2017). On the other hand, as we have seen, focusing on a single, local measure can lead us to neglect some other factor or confound, thereby rendering the results from that measure at best incomplete, and at worst meaningless. As such, it seems as though a balance between these two concerns is required. The approach taken by researchers is generally to use multiple measures, but not correct for multiple comparisons across measures. We do not intend to answer what will likely be a long and extensive debate on this topic here, but we can advise that researchers seek to use as few measures as possible when analyzing their results, focusing on the minimum required to assess or check their predictions. Ideally, researchers would pre-register their analysis plan prior to analysis. Pre-registration could both protect against questions of cherry-picking results, and simplify the analyses and quantity of data to report. Above all else, it is vital that, whatever measures are used in a given study, researchers very much focus on analyzing measures that clearly and accurately address the theoretical questions being asked in that study.

One further point for consideration on the issue of which measures to use is the tendency for new and highly specific measures to be “created” for a single study. Often, based on the experimental design, researchers will develop a new dependent variable to focus on. Whilst this is of course an entirely reasonable approach to take, there is a real and often unacknowledged, danger in doing so. Specifically, when using a newly created measure, one can, without realizing, develop cleaning or inferential approaches that are confounded compared to measures that have been used for decades and been tried and tested extensively. As noted by Flake and Fried (2020), we would recommend caution be taken when developing new and specific measures for different studies and to use standard, already-existing measures wherever possible.

## General discussion: Recommendations and conclusions

We have now concluded our walkthrough of a visual search and eye-movement study, highlighting various pitfalls along the way. We noted five major pitfalls, but also discussed a variety of other issues involving reporting of methods, methodology, terminology, data cleaning, and analytical decisions that could prove problematic to researchers. We have attempted to provide a snapshot of how widespread these pitfalls might be within the existing literature, although we do believe that a more formal systematic review of the existing literature would be valuable in helping to understand how prevalent the pitfalls are in the literature. Our hope is that the discussion and information provided here will be of value both to researchers who are new to the study of visual search and eye movements as well as researchers who have been working in the field for some time. It is certainly the case that, when writing this tutorial review, we reflected on our own work and found that we had fallen prey to pitfalls in numerous places (as can be seen in our discussions regarding Godwin et al. (2015) throughout this review, as well as the papers discussed for our *Brief review*, several of which share co-authors with this review).

We have summarized the potential pitfalls and their likely impact upon datasets and conclusions in Table 2. It is worth noting that these are the *likely* but not *certain* impacts upon datasets and conclusions. As one might expect, the reality is that without direct checking of datasets it is impossible to be certain whether the conclusions can still be supported after checking for the pitfalls. Overall, zero papers in our sample were able to avoid all pitfalls.

**Table 2:** Pitfalls and their likely outcomes

Pitfall	Likely outcome	*Brief review* sample that avoided this pitfall
*#1 Failing to consider visual similarity when generating predictions or designing a study*	Finding evidence of significant differences that would be expected from visual similarity alone	85%
*#2 Failing to consider coarse-to-fine behavior when generating predictions*	Finding evidence of significant differences that would be expected from coarse-to-fine behavior alone	6.25%
*#3 Failing to ensure that participants begin by fixating the desired starting point of the search display*	Concluding that measures of time to fixate certain objects can be trusted without more detailed cleaning	35%
*#4 Failure to consider and clean eye-movement data based on event contingencies*	Distortion of measures based upon fixation durations	5%
*#5 Failing to calculate appropriate values for each participant*	Distortion of all means, particularly those based on fixation durations and saccade amplitudes. The distortion will likely increase the means	0%

Of course, some readers of this tutorial review may disagree that these pitfalls threaten conclusions drawn in published research and/or that any effects will survive re-analysis conducted under consideration of these pitfalls. That will undoubtedly be true in many cases. Yet, even if the conclusions remain unchanged after accounting for the pitfalls, we still believe that it is important for researchers to provide a veridical and accurate set of analyses after proper processing and cleaning, even if their conclusions remain the same. Worse still is the fact that even if a re-analysis is conducted, different groups of researchers may not necessarily agree upon how to re-analyze a given dataset, with each pitfall or minor issue discussed here being a potential source of contention. After all, different laboratories and researchers all have their own set of entrenched approaches, traditions, and beliefs.

Rather than spend the coming years debating whether to treat fixations as outliers if they are, say, less than 80 ms versus less than 70 ms (to take but one contrived example), it is our belief that a more productive approach would be to simply map out all reasonable analyses that could be conducted upon a given dataset. Under this approach, called a *multiverse* analysis, a researcher conducts not one analysis, but many (e.g., see Steegen et al., 2016). Precedent for this already exists in the study of visual search and eye movements. We noted when discussing data filtering that some researchers already examine datasets twice: once treating fixations as landing on objects only if they fell directly upon those objects and a second time using a distance threshold limit that includes a larger sample of the fixations in the study. This approach is a (small) form of multiverse analysis (i.e., one with two “universes” in). The benefit of multiverse analyses is that they make transparent the many possible and different reasonable data processing pipelines that can be adopted when filtering and cleaning complex datasets such as those recorded as part of visual search and eye-movement experiments. Rather than arbitrarily removing certain outliers or making claims regarding the best way to process a dataset, a researcher can *show* that their approach is a reasonable one that is not contingent upon them sub-selecting a very specific set of values that, as fortune has it, fits their predictions.

The choices made as part of the data-filtering process are often referred to as a *garden of forking paths*, with each decision about filtering the data leading to an explicit, or perhaps even implicit, fork in the path (Gelman & Loken, 2014). We can begin to use this philosophy, and the multiverse analysis approach that comes with it, to highlight just how easily conclusions about a dataset can change. To take one example, we close this point by posing a final question of the dataset from Godwin et al. (2015): Was there a difference in how long participants spent fixating each object between the two experiments? In Experiment 1, participants searched for dissimilar targets; in Experiment 2, they searched for similar targets. We might pose this question given that searching for two similar targets is known to be easier than searching for two dissimilar targets (Hout & Goldinger, 2015; Menneer et al., 2012; Mestry et al., 2017), so perhaps we might expect fixation durations to be longer for Experiment 1 than Experiment 2. Moreover, we chose this measure in part given our earlier discussion regarding the concern that total time spent fixating objects might be confounded and that single fixation durations may be a better indicator of total processing time (per Orquin & Holmqvist, 2018).

**Fig. 12:**
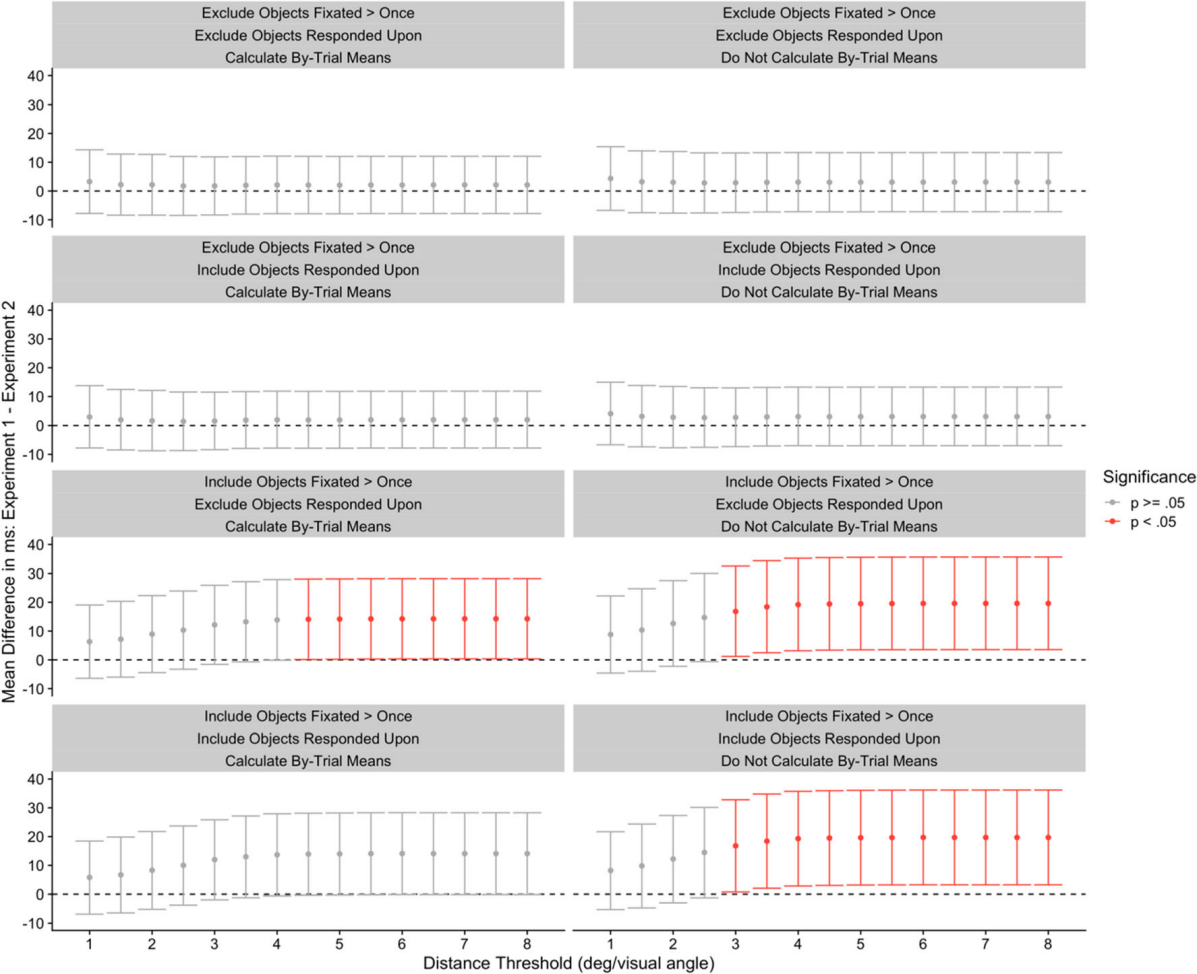
Results of multiverse analysis

With that in mind, we conducted a set of between-subjects *t*-tests: 120 such *t*-tests, in fact. The results of these are presented in Fig. 12. They present a set of reasonable analyses that researchers might conduct to address the question posed. On the x-axis is the distance limit – that is, how close to a given object a fixation needs to be in order for it to be considered as having fallen on that object. We set the upper value of this to 8° of visual angle to simulate the approach taken wherein the nearest fixation to an object, regardless of distance, is assigned as landing on that object. We chose the value of 8° of visual angle because all fixations in the dataset fell within that distance from an object. The panels in Fig. 12 present a different combination of just three filters:

- Whether or not a given object was fixated only once (to avoid calculating the potentially confounded measure of local total time fixating).
- Whether or not an object was fixated alongside a response (in order to consider event contingencies associated with responses; see pitfall #4).
- Whether or not by-trial means were calculated (to avoid inflating means; see pitfall #5).

In all cases, when the dataset included objects that were fixated only once, the outcome was not significant (top two rows). This was not the case when the restriction for objects to be fixated only once was lifted. Without this restriction, as can be seen in the lower two rows of Fig. 12, a number of the *t*-tests produced a significant result. Based upon our *Brief review*, we believe that a great deal of the existing literature falls into the lower two panels on the right-hand side of Fig. 12. Unfortunately for these, it happens to be the easiest set of conditions that can produce a significant result. Here, the high distance threshold (which mirrors situations where the “nearest-object-to-fixation” approach is taken), coupled with the fact that fixations that coincided with a response have been included, plus the lack of calculating by-trial means has combined to distort the values to a point where the majority of the comparisons yield a significant result. Across the entire multiverse here, 25% of the comparisons yielded a significant result, yet none were significant in the top left panel, which is, when using stricter filters, where we would recommend researchers focus their efforts when it comes to filtering and cleaning datasets. In that top left panel, several pitfalls are accounted for (at least, those that were considered here). Overall, the *p* value from the multiverse analysis was *p* = .75, since 75% of the comparisons were not significant.^11^

Of course, the multiverse analysis approach is helpful primarily when it comes to processing and analyzing datasets, and it does little to stem the tide of pitfalls that arise before a single eye movement is recorded or before predictions are made and experimental design decisions are determined. On this front, it is difficult to provide a clear or concrete set of recommendations because much of the issue here is that each experiment carries with it a unique combination of predictions and stimuli such that a ‘one size fits all’ set of recommendations is not readily forthcoming. Perhaps our best recommendation is to encourage researchers to carefully consider all of the pitfalls discussed, as well as others in this emerging literature on pitfalls associated with eye-movement studies. And, more than that, we would encourage readers to provide as much detail and justification as they can regarding the setup of their eye-trackers, their data-filtering approaches, and their fundamental definitions (e.g., what they define as a fixation or a saccade). We believe it is unlikely that a consensus on terminology will ever emerge at sufficient scale so as to make a difference (or at least, this would take many years to emerge). For now, it would at least be helpful for researchers to provide a clear lexicon of the fundamental building blocks that underlie their conclusions, explicitly defining what they mean when they invoke each dependent variable, for example.

We do not mean to sound pessimistic here, for we are optimistic about changes and the challenges that lay ahead in the future for studies of visual search and eye movements. More and more, researchers are beginning to become aware of, and openly discuss, pitfalls much like those that have been described here, including in the study of eye-movement behavior. The first step to making improvements or refinements will naturally be recognizing that there is a problem, and the second step will be to devise solutions to tackle any problems that are identified. Solutions could include broader sharing of data to permit an assessment of the pitfalls and their effect on experiments and a widening of access to different software tools (e.g., the eyeTrackR package by Godwin & Muhl-Richardson, 2019, can help to resolve some of the pitfalls discussed here).

Through discussions of these and other pitfalls, we are hopeful that researchers will seek to avoid them because, in many cases, we suspect that researchers have fallen prey to the pitfalls simply because they were not aware that the pitfalls existed. And why should we expect those pitfalls to be well understood? Many of the pitfalls are avoided by more experienced researchers in an opaque manner, with solutions to the pitfalls being woven into experimental designs and analytic procedures that were not discussed or made explicit in published work. Within published research is enshrined the key elements of what is required to replicate an experiment, but so rarely is information given within *Methods* or *Results* sections of papers regarding *why* certain methods or analytic choices were made. Becoming cognizant of that fact has been perhaps one of the greatest lessons we have learned whilst reflecting upon our own published work in this area when writing this tutorial review: Put simply, we should have been clearer about why we set up an experiment in a certain way, why datasets were cleaned in a particular manner, and we should have been up-front about what were, in all honesty, arbitrary decisions in some cases.

In 1895, not long before Delabarre (1898) published an account of using plaster-of-Paris connected to a piece of fiber to record eye-movement behavior, Joseph Malins wrote what is now a famous poem entitled *The Ambulance Down in the Valley.* This allegorical poem tells the tale of a cliffside. From this cliffside, many people fall to their demise, and a debate is struck amongst the townsfolk: Some argue for the construction of a fence around the cliff-edge to protect others from falling; others argue in favor of setting up an ambulance to pick up unfortunate individuals after they have fallen. This poem, and its debate, resonates with much of the content of this tutorial review. From our perspective, we would recommend the construction of as many fences upon the cliff and the recruitment of as many ambulances at the bottom of the cliff as possible. By better understanding where researchers can fall prey to pitfalls before a study even begins, we can put up more fences to protect studies before they fall; by refining and developing our analytic approaches, we can recruit more ambulances at the bottom of the cliff to protect well-formed and conducted studies from falling prey to traps at the data analysis stage of the research cycle.

## Supplementary Information

ESM 1 (HTML 1214 kb)

## Footnotes

1 Mean fixation duration was 162 ms, with a standard deviation of 14.43 ms, a minimum of 141.6 ms and a maximum of 196 ms.

2 This 2015 paper was not captured as part of our *Brief review* since it was first published online in 2014.

3 We refer to this study as “exploratory” because the sample size of five participants did not permit statistical analyses (three of the participants were authors on the published paper).

4 Not all trackers use a validation procedure, but it is useful in aiding the researcher in monitoring the calibration accuracy online, re-calibrating when necessary.

5 An event based on personal experience with data collection.

6 These are drawn from an unpublished visual search study. Sample data are not available from Godwin et al. (2015) due to poor data-archiving practices.

7 We are not trying to be critical of the methods used in this study, especially since the authors went to such great lengths to ensure that their data were of the highest quality.

8 One other option, often made available by eye-tracking software, is to “trim” the fixation durations at the point an event occurs. For example, a researcher could trim a fixation duration on a target such that the duration ends when a response button is pressed. We regard this as a potentially dangerous procedure since it will necessarily involve distorting the true fixation durations and in turn, shift those durations to values that are not reflective of actual fixation durations.

9 Some measures are not susceptible to this issue, but often we found when conducting our *Brief review* that it was difficult to determine one way or another whether contingencies had been considered and filtered appropriately. For that reason, coupled with the fact that correct filtering is extremely important, we took a strict approach here and sought to determine whether a consideration of event contingencies and the time period under examination was *explicitly* noted.

10 It is worth noting that the distance-based approach can fail when overlapping or asymmetric objects are used in the search displays, because their center locations can be skewed by their asymmetric shape, as was the case in a study conducted by Godwin et al. (2017).

11 Our multiverse approach here is intended to serve as a brief illustration and has not completed all of the analytic steps required for an analysis of this nature. However, we hope that the illustration serves as a useful introduction.
