# Supplementary material for: Avoiding potential pitfalls in visual search and eye-movement experiments: A tutorial review
Source: Atten Percept Psychophys. 2021 Jun 4;83(7):2753–83. doi: 10.3758/s13414-021-02326-w (PMC8460493; doi:10.3758/s13414-021-02326-w)
Supplement: Supplementary file 1 — (HTML 1214 kb) [file 13414_2021_2326_MOESM1_ESM.html]

Brief Review of Previous Visual Search and Eye Movements Papers


# Brief Review of Previous Visual Search and Eye Movements Papers

Here we present the results of our Brief Review of previous visual search and eye movements papers.

# Inclusions and General Metrics

# Choosing the Right Predictions

# Choosing your Eye-tracker

# Setting up and Calibrating your Eye-tracker

# Recording Eye Movement Behavior During a Visual Search Trial

# Cleaning Your Data

# Analyses

# Global Measures

# Local Measures

# References

Adler, S. A., & Gallego, P. (2014). Search asymmetry and eye movements in infants and adults. *Attention, Perception, & Psychophysics*, *76*(6), 1590–1608. https://doi.org/10.3758/s13414-014-0667-6

Anderson, G. M., & Humphreys, G. W. (2015). Top-down expectancy versus bottom-up guidance in search for known color-form conjunctions. *Attention, Perception, & Psychophysics*, *77*(8), 2622–2639. https://doi.org/10.3758/s13414-015-0960-z

Azizi, E., Abel, L. A., & Stainer, M. J. (2017). The influence of action video game playing on eye movement behaviour during visual search in abstract, in-game and natural scenes. *Attention, Perception, & Psychophysics*, *79*(2), 484–497. https://doi.org/10.3758/s13414-016-1256-7

Barrett, D. J. K., & Zobay, O. (2020). Concurrent evaluation of independently cued features during perceptual decisions and saccadic targeting in visual search. *Attention, Perception, & Psychophysics*, *82*(3), 966–984. https://doi.org/10.3758/s13414-019-01854-w

Brams, S., Ziv, G., Hooge, I. T. C., Levin, O., De Brouwere, T., Verschakelen, J., Dauwe, S., Williams, A. M., Wagemans, J., & Helsen, W. F. (2020). Focal lung pathology detection in radiology: Is there an effect of experience on visual search behavior? *Attention, Perception, & Psychophysics*, *82*(6), 2837–2850. https://doi.org/10.3758/s13414-020-02033-y

Casteau, S., & Smith, D. T. (2020). On the link between attentional search and the oculomotor system: Is preattentive search restricted to the range of eye movements? *Attention, Perception, & Psychophysics*, *82*(2), 518–532. https://doi.org/10.3758/s13414-019-01949-4

Chabal, S., Schroeder, S. R., & Marian, V. (2015). Audio-visual object search is changed by bilingual experience. *Attention, Perception, & Psychophysics*, *77*(8), 2684–2693. https://doi.org/10.3758/s13414-015-0973-7

Chetverikov, A., Kuvaldina, M., MacInnes, W. J., Jóhannesson, Ó. I., & Kristjánsson, Á. (2018). Implicit processing during change blindness revealed with mouse-contingent and gaze-contingent displays. *Attention, Perception, & Psychophysics*, *80*(4), 844–859. https://doi.org/10.3758/s13414-017-1468-5

Christie, G. J., Spalek, T. M., & McDonald, J. J. (2018). Salience drives overt selection of two equally relevant visual targets. *Attention, Perception, & Psychophysics*, *80*(6), 1342–1349. https://doi.org/10.3758/s13414-018-1555-2

Cimminella, F., Sala, S. D., & Coco, M. I. (2020). Extra-foveal Processing of Object Semantics Guides Early Overt Attention During Visual Search. *Attention, Perception, & Psychophysics*, *82*(2), 655–670. https://doi.org/10.3758/s13414-019-01906-1

Couperus, J. W., & Quirk, C. (2015). Visual search and the N2pc in children. *Attention, Perception, & Psychophysics*, *77*(3), 768–776. https://doi.org/10.3758/s13414-015-0833-5

Decroix, J., & Kalénine, S. (2019). What first drives visual attention during the recognition of object-directed actions? The role of kinematics and goal information. *Attention, Perception, & Psychophysics*, *81*(7), 2400–2409. https://doi.org/10.3758/s13414-019-01784-7

Draschkow, D., & Võ, M. L.-H. (2016). Of “what” and “where” in a natural search task: Active object handling supports object location memory beyond the object’s identity. *Attention, Perception, & Psychophysics*, *78*(6), 1574–1584. https://doi.org/10.3758/s13414-016-1111-x

Eymond, C., Cavanagh, P., & Collins, T. (2019). Feature-based attention across saccades: Pop-out in color search is spatiotopic. *Attention, Perception, & Psychophysics*, *81*(1), 85–97. https://doi.org/10.3758/s13414-018-1597-5

Fabius, J. H., Schut, M. J., & Van der Stigchel, S. (2016). Spatial inhibition of return as a function of fixation history, task, and spatial references. *Attention, Perception, & Psychophysics*, *78*(6), 1633–1641. https://doi.org/10.3758/s13414-016-1123-6

Finlayson, N. J., & Grove, P. M. (2015). Visual search is influenced by 3D spatial layout. *Attention, Perception, & Psychophysics*, *77*(7), 2322–2330. https://doi.org/10.3758/s13414-015-0924-3

Gao, Y.-Y., Schneider, B., & Li, L. (2017). The effects of the binocular disparity differences between targets and maskers on visual search. *Attention, Perception, & Psychophysics*, *79*(2), 459–472. https://doi.org/10.3758/s13414-016-1252-y

Grossberg, S. (2019). The resonant brain: How attentive conscious seeing regulates action sequences that interact with attentive cognitive learning, recognition, and prediction. *Attention, Perception, & Psychophysics*, *81*(7), 2237–2264. https://doi.org/10.3758/s13414-019-01789-2

Hannus, A., Bekkering, H., & Cornelissen, F. W. (2020). Preview of partial stimulus information in search prioritizes features and conjunctions, not locations. *Attention, Perception, & Psychophysics*, *82*(1), 140–152. https://doi.org/10.3758/s13414-019-01841-1

Higuchi, Y., Inoue, S., Endo, T., & Kumada, T. (2019). Task-irrelevant optic flow guides attention in visual search. *Attention, Perception, & Psychophysics*, *81*(5), 1327–1345. https://doi.org/10.3758/s13414-018-01646-8

Hilchey, M. D., Leber, A. B., & Pratt, J. (2018). Testing the role of response repetition in spatial priming in visual search. *Attention, Perception, & Psychophysics*, *80*(6), 1362–1374. https://doi.org/10.3758/s13414-018-1550-7

Hollingworth, A., & Matsukura, M. (2019). Feature-based guidance of attention during post-saccadic selection. *Attention, Perception, & Psychophysics*, *81*(6), 1822–1835. https://doi.org/10.3758/s13414-019-01719-2

Hout, M. C., & Goldinger, S. D. (2015). Target templates: The precision of mental representations affects attentional guidance and decision-making in visual search. *Attention, Perception, & Psychophysics*, *77*(1), 128–149. https://doi.org/10.3758/s13414-014-0764-6

Hout, M. C., Robbins, A., Godwin, H. J., Fitzsimmons, G., & Scarince, C. (2017). Categorical templates are more useful when features are consistent: Evidence from eye movements during search for societally important vehicles. *Attention, Perception, & Psychophysics*, *79*(6), 1578–1592. https://doi.org/10.3758/s13414-017-1354-1

Hulleman, J., Lund, K., & Skarratt, P. A. (2020). Medium versus difficult visual search: How a quantitative change in the functional visual field leads to a qualitative difference in performance. *Attention, Perception, & Psychophysics*, *82*(1), 118–139. https://doi.org/10.3758/s13414-019-01787-4

Kruijne, W., & Meeter, M. (2016). Implicit short- and long-term memory direct our gaze in visual search. *Attention, Perception, & Psychophysics*, *78*(3), 761–773. https://doi.org/10.3758/s13414-015-1021-3

Lewis, J. E., & Neider, M. B. (2015). Fixation Not Required: Characterizing Oculomotor Attention Capture for Looming Stimuli. *Attention, Perception, & Psychophysics*, *77*(7), 2247–2259. https://doi.org/10.3758/s13414-015-0950-1

Lin, Z., Lu, Z.-L., & He, S. (2016). Decomposing experience-driven attention: Opposite attentional effects of previously predictive cues. *Attention, Perception, & Psychophysics*, *78*(7), 2185–2198. https://doi.org/10.3758/s13414-016-1101-z

Lleras, A., Wang, Z., Ng, G. J. P., Ballew, K., Xu, J., & Buetti, S. (2020). A target contrast signal theory of parallel processing in goal-directed search. *Attention, Perception, & Psychophysics*, *82*(2), 394–425. https://doi.org/10.3758/s13414-019-01928-9

MacInnes, W. J., Hunt, A. R., Hilchey, M. D., & Klein, R. M. (2014). Driving forces in free visual search: An ethology. *Attention, Perception, & Psychophysics*, *76*(2), 280–295. https://doi.org/10.3758/s13414-013-0608-9

Maturi, K. S., & Sheridan, H. (2020). Expertise effects on attention and eye-movement control during visual search: Evidence from the domain of music reading. *Attention, Perception, & Psychophysics*, *82*(5), 2201–2208. https://doi.org/10.3758/s13414-020-01979-3

Mayer, K. M., Thornton, I. M., & Vuong, Q. C. (2020). Comparable search efficiency for human and animal targets in the context of natural scenes. *Attention, Perception, & Psychophysics*, *82*(3), 954–965. https://doi.org/10.3758/s13414-019-01901-6

Meghanathan, R. N., Nikolaev, A. R., & Leeuwen, C. van. (2019). Refixation patterns reveal memory-encoding strategies in free viewing. *Attention, Perception, & Psychophysics*, *81*(7), 2499–2516. https://doi.org/10.3758/s13414-019-01735-2

Mine, C., & Saiki, J. (2018). Pavlovian reward learning elicits attentional capture by reward-associated stimuli. *Attention, Perception, & Psychophysics*, *80*(5), 1083–1095. https://doi.org/10.3758/s13414-018-1502-2

Morgan, M. J., & Solomon, J. A. (2020). A visual search asymmetry for relative novelty in the visual field based on sensory adaptation. *Attention, Perception, & Psychophysics*, *82*(3), 938–943. https://doi.org/10.3758/s13414-019-01943-w

Ng, G. J. P., Lleras, A., & Buetti, S. (2018). Fixed-target efficient search has logarithmic efficiency with and without eye movements. *Attention, Perception, & Psychophysics*, *80*(7), 1752–1762. https://doi.org/10.3758/s13414-018-1561-4

Niimi, R. (2020). Interacting hands draw attention during scene observation. *Attention, Perception, & Psychophysics*, *82*(3), 1088–1098. https://doi.org/10.3758/s13414-019-01881-7

Nissens, T., & Fiehler, K. (2020). The attractiveness of salient distractors to reaching movements is task dependent. *Attention, Perception, & Psychophysics*, *82*(5), 2502–2515. https://doi.org/10.3758/s13414-020-01984-6

Proctor, R. W., & Healy, A. F. (2020). Visual selection and response selection without effector selection in tasks with circular arrays. *Attention, Perception, & Psychophysics*. https://doi.org/10.3758/s13414-020-02116-w

Ratiu, I., Hout, M. C., Walenchok, S. C., Azuma, T., & Goldinger, S. D. (2017). Comparing visual search and eye movements in bilinguals and monolinguals. *Attention, Perception, & Psychophysics*, *79*(6), 1695–1725. https://doi.org/10.3758/s13414-017-1328-3

Retell, J. D., Venini, D., & Becker, S. I. (2015). Oculomotor Capture by New and Unannounced Color Singletons during Visual Search. *Attention, Perception, & Psychophysics*, *77*(5), 1529–1543. https://doi.org/10.3758/s13414-015-0888-3

Smith, M. K., & Grabowecky, M. (2020). Exogenous Orientation of Attention to the Center of Mass in a Visual Search Task. *Attention, Perception, & Psychophysics*, *82*(2), 729–738. https://doi.org/10.3758/s13414-019-01908-z

Solman, G. J. F., Hickey, K., & Smilek, D. (2014). Comparing target detection errors in visual search and manually-assisted search. *Attention, Perception, & Psychophysics*, *76*(4), 945–958. https://doi.org/10.3758/s13414-014-0641-3

Stefani, M., Sauter, M., & Mack, W. (2020). Delayed disengagement from irrelevant fixation items: Is it generally functional? *Attention, Perception, & Psychophysics*, *82*(2), 637–654. https://doi.org/10.3758/s13414-019-01926-x

Verghese, A., Mattingley, J. B., Palmer, P. E., & Dux, P. E. (2018). From eyes to hands: Transfer of learning in the Simon task across motor effectors. *Attention, Perception, & Psychophysics*, *80*(1), 193–210. https://doi.org/10.3758/s13414-017-1427-1

Walenchok, S. C., Hout, M. C., & Goldinger, S. D. (2016). Implicit object naming in visual search: Evidence from phonological competition. *Attention, Perception, & Psychophysics*, *78*(8), 2633–2654. https://doi.org/10.3758/s13414-016-1184-6

Wang, F., Sun, J., Sun, P., Weidler, B. J., & Abrams, R. A. (2017). Influence of simple action on subsequent manual and ocular responses. *Attention, Perception, & Psychophysics*, *79*(2), 389–395. https://doi.org/10.3758/s13414-017-1280-2

Zhang, H., & Houpt, J. W. (2020). Exaggerated prevalence effect with the explicit prevalence information: The description-experience gap in visual search. *Attention, Perception, & Psychophysics*, *82*(7), 3340–3356. https://doi.org/10.3758/s13414-020-02045-8

Zhao, F., & Ren, Y. (2020). Revisiting contextual cueing effects: The role of perceptual processing. *Attention, Perception, & Psychophysics*, *82*(4), 1695–1709. https://doi.org/10.3758/s13414-019-01962-7
